# Can breastfeeding promote child health equity? A comprehensive analysis of breastfeeding patterns across the developing world and what we can learn from them

**DOI:** 10.1186/1741-7015-11-254

**Published:** 2013-12-04

**Authors:** Thomas J Roberts, Emily Carnahan, Emmanuela Gakidou

**Affiliations:** 1 Stanford University School of Medicine, Stanford, CA 94305, USA; 2 PATH, Seattle, WA 98121, USA; 3 The Institute for Health Metrics and Evaluation (IHME), University of Washington, Seattle, WA 98121, USA

**Keywords:** Breastfeeding, Health inequity, Child health, Global burden of disease, Infant feeding

## Abstract

**Background:**

In 2010 more than 7.7 million children died before their fifth birthday. Over 98% of these deaths occurred in developing countries, and recent estimates have attributed hundreds of thousands of these deaths to suboptimal breastfeeding.

**Methods:**

This study estimated prevalence of suboptimal breastfeeding for 137 developing countries from 1990 to 2010. These estimates were compared against WHO infant feeding recommendations and combined with effect sizes from existing literature to estimate associated disease burden using a standard comparative risk assessment approach. These prevalence estimates were disaggregated by wealth quintile and linked with child mortality rates to assess how improved rates of breastfeeding may affect child health inequalities.

**Results:**

In 2010, the prevalence of exclusive breastfeeding ranged from 3.5% in Djibouti to 77.3% in Rwanda. The proportion of child Disability Adjusted Life Years (DALYs) attributable to suboptimal breastfeeding is 7.6% at the global level and as high as 20.2% in Swaziland. Suboptimal breastfeeding is a leading childhood risk factor in all developing countries and consistently ranks higher than water and sanitation. Within most countries, breastfeeding prevalence rates do not vary considerably across wealth quintiles.

**Conclusions:**

Breastfeeding is an effective child health intervention that does not require extensive health system infrastructure. Improvements in rates of exclusive and continued breastfeeding can contribute to the reduction of child mortality inequalities in developing countries.

## Background

Suboptimal breastfeeding has been linked with numerous adverse child health outcomes including increased incidence of diarrhea and pneumonia and increased mortality [1-4]. However, breastfeeding prevalence in nearly every country of the world continues to fall significantly short of levels recommended by the World Health Organization (WHO) and other infant nutrition experts. *The Lancet’s* Child Survival Series estimated that 13% of all child deaths in low-income countries could be prevented if breastfeeding prevalence was increased to optimal levels [5]. The importance of breastfeeding was further supported by two recent comparative risk assessments. The Global Burden of Diseases, Injuries, and Risk Factors Study 2010 (GBD 2010) ranked suboptimal breastfeeding as the second largest risk factor for children under five, accounting for 47.5 million Disability Adjusted Life Years (DALYs) lost in 2010. This study also found the highest proportion of disease burden associated with suboptimal breastfeeding in regions such as Sub-Saharan Africa where childhood morbidity and mortality are highest [2]. *The Lancet’s* 2013 Maternal and Child Nutrition Series also found suboptimal breastfeeding to be responsible for 804,000 deaths in 2011, 11.6% of total deaths of children under five years old [1-4,6].

In recent years WHO has issued several rounds of breastfeeding recommendations. The most recent major revisions to the recommendations were issued in 2001 and include three key components: timely initiation (within one hour of birth) of breastfeeding, exclusive breastfeeding up to the age of six months, and continued breastfeeding through 24 months [5,7]. Many national and international surveys have been adapted to include questions on these elements, and compiling these data to assess levels and trends in breastfeeding patterns can help evaluate child health interventions. However, producing estimates of prevalence and associated disease burden directly from these surveys ignores temporal patterns and can lead to inconsistencies across countries. The need for consistent estimates of breastfeeding prevalence and trends across all countries was recently highlighted in *The Lancet’s* 2013 Maternal and Child Nutrition Series [6].

Recent shifts in global health priority setting that have placed increasing emphasis on health equity have increased the appeal of interventions that can more easily reach populations with limited access to health infrastructure. It is well documented that child health interventions, such as antenatal care, vaccinations and improved management of diarrhea and pneumonia, exhibit significant wealth-related inequality. When these interventions are scaled-up, they tend to reach wealthier segments of society first and increase child health inequalities until they are able to achieve very high coverage rates [8]. With the increasing focus on equity, interventions such as breastfeeding promotion, which have proven child health effects and can be implemented independent of health infrastructure, will become increasingly appealing for their ability to improve child health while also promoting equity.

In recent years, there have been many well-documented national and international programs to improve breastfeeding practices. WHO and UNICEF have issued promotion guidelines as part of programs such as the Baby Friendly Hospital Initiative and organizations such as USAID have invested heavily in international breastfeeding promotions [9,10]. Many of these efforts have been able to increase breastfeeding prevalence significantly, and breastfeeding promotion is widely considered one of the most cost-effective child health interventions available [11-13]. However, some programs have had mixed results, and even countries with successful programs still have breastfeeding prevalence rates far below the levels recommended by WHO [10,14,15]. One of the issues behind the varied results of these programs is the complexity of attitudes towards breastfeeding. A woman’s decision to breastfeed is multifactorial, and it has been widely shown that different traits and behaviors have been associated with breastfeeding in different contexts [16-19]. Variation of these factors may contribute to the limitations reported from some breastfeeding promotion projects. These realities are creating increasing urgency to understand what causes breastfeeding promotion interventions to work and how they can be successfully implemented in more contexts.

The absence of a comprehensive time series to analyze patterns across countries and across wealth quintiles within countries limits our ability to understand variations in indicators associated with breastfeeding and thus limits the ability of program planners to tailor interventions towards target populations. In this paper we produce a comprehensive time series across countries and explore trends in breastfeeding across wealth quintiles.

## Methods

### Data inputs

The first step in this analysis was to identify the most appropriate breastfeeding exposures to measure. The ideal measures would accurately portray the state of breastfeeding practices relative to the current WHO recommendations, facilitate the inclusion of as many data sources as possible and be easily decipherable by a large audience. After weighing these three factors, the following measures were selected: breastfeeding initiation within 24 hours of birth; exclusive, predominant, and partial breastfeeding prevalence in children under six months of age; and continued breastfeeding prevalence in children between 6 and 24 months of age. For this analysis, the definitions used for these variables were consistent with WHO definitions [7].

A comprehensive search was conducted to identify all available data, both published and unpublished, on breastfeeding patterns in developing countries. These data were evaluated in four areas: how well they measured the outcomes of interest; the national representativeness of the study population; the quality of the data collection methods; and the quality of the data analysis or the availability of microdata. In total, nearly 400 data sources were located and found to be of sufficient quality for inclusion with several major surveys contributing substantial amounts of data to this analysis. The breakdown of data sources is shown in Table 1.

**Table 1:** Data sources included in analysis

**Survey type**	**Breastfeeding initiation**	**Exclusive/predominant/partial breastfeeding**	**Continued breastfeeding**
DHS	138	192	197
MICS	4	78	78
PAPFAM	6	6	6
RHS	1	23	26
Country-specific	12	34	56
Total	161	333	363

DHS, Demographic and Health Surveys; MICS, Multiple Indicator Cluster Survey; PAPFAM, Pan-Arab Project for Family Health; RHS, Reproductive Health Survey.

Data included in the analysis covered a 25-year period during which WHO recommendations for breastfeeding practices and breastfeeding data collection methods underwent several revisions. As a result, data included in this paper came from several different data collection methods and reporting mechanisms. When microdata were available, we estimated prevalence using methods consistent with current WHO definitions [7]. When microdata were not available, we estimated correction factors to convert non-standard estimates to the measures reported in this paper. The correction factors were estimated directly from the Demographic and Health Surveys (DHS) data.

### Prevalence estimates

A three-step statistical model was used to generate a complete set of breastfeeding prevalence estimates for 137 developing countries from 1990 to 2010, including uncertainty.

These models relied on having a set of covariates that were related to breastfeeding patterns. To identify relationships with covariates at the country level, correlation coefficients were calculated between the prevalence data for each indicator and the potential covariates. The covariates most closely correlated with the breastfeeding indicators were selected to be used in the first step of the model.

The first step of the model was an ordinary least squares (OLS) regression of each breastfeeding outcome. The following predictor variables were used: 1) mean years of education of women of reproductive age; 2) gross domestic product (GDP) per capita; 3) underweight (>2 SD below mean weight for age); and 4) total fertility rate. The models can be summarized as follows:

$$\begin{array}{l} \mathrm{BFva}r_{i,t}=\beta_{0}+\beta_{1}\mathrm{Ed}u_{i,t}+\beta_{1}\mathrm{GD}P_{i,t}+\beta_{1}\mathrm{Wf}A_{i,t}\\ \;+\beta_{1}\mathrm{TF}R_{i,t}+\epsilon_{i,t} \end{array}$$

Given that a lot of the variation in breastfeeding prevalence was not explained by the modeled covariates, the second step employed a spatial-temporal regression using the residuals from the OLS regression. We performed a locally weighted regression that allows residuals nearby in space and time to have more weight than those farther away. Spatial relationships were based on regions defined for the GBD 2010 [20]. In order to prevent subnational or seasonally-biased data from unduly influencing the national trends, we down-weighted the total contribution of any non-nationally-representative data points to 10% while the contribution of nationally-representative data comprised the remaining 90%. The predicted residuals are added on to the predictions from the OLS regression.

The final step was a Gaussian Process Regression (GPR) that uses the results from the spatial-temporal regression as the mean function and draws from a multinomial distribution, based on the uncertainty in the data and in the prior, to generate 1,000 draws of a posterior distribution. These draws were used to calculate the final mean and confidence interval estimates.

The three step modeling process applied here, including modeling parameters, has been described in detail elsewhere [21]. Because a literature search for relationships between breastfeeding behaviors and possible covariates did not reveal any strong, consistent relationships that could be used to reliably predict breastfeeding prevalence rates in the absence of data, our model parameters were selected to place greater weight on the existing data over the prior mean function in the spatial-temporal regressions and GPR. As a result, our models were not sensitive to the covariates used in the first stage OLS regression.

### Inequalities in prevalence

Where possible, breastfeeding and wealth information were linked to explore breastfeeding prevalence by wealth quintile. There were 141 Multiple Indicator Cluster Survey (MICS) and DHS datasets where breastfeeding and income/asset questions were linked at the individual level. MICS datasets contain a calculated variable for wealth quintile, and this variable was linked directly with the breastfeeding indicators.

For the DHS datasets, we used a two-step Bayesian latent variable model to calculate the permanent income of each household. The first step of this method used a probit model to establish a cutpoint for each asset. We utilized all assets that are distributed as normal or reverse-normal goods as indicative of wealth. In the second step, the covariates and coefficients from the probit model were used to generate a prior for each household. This prior was then updated through a dichotomous hierarchical ordered probit model to generate permanent income estimates for each household. These methods are described in detail elsewhere [22]. After the household permanent income was estimated, all households were ordered and assigned to the appropriate wealth quintile.

Breastfeeding prevalence was calculated for each quintile. In all countries with at least three data points, prevalence trends in each quintile were compared to determine how changes in breastfeeding prevalence varied across wealth quintiles.

### Attributable burden

Comparative risk assessment methods were used to produce estimates of the quantity of childhood morbidity and mortality attributable to suboptimal breastfeeding, while holding other independent factors unchanged. These methods have been described in detail elsewhere [2].

Attributable burden was calculated with reference to a counterfactual distribution of exposure, known as the theoretical-minimum-risk exposure distribution. For this analysis, the theoretical-minimum-risk exposure distribution was set at 100% compliance with the current WHO recommendations, that is, that 100% of children are exclusively breastfed until six months of age and have continued breastfeeding through 24 months. This value was chosen based on the advice of experts who indicated that almost all women are able to initiate breastfeeding with proper coaching.

Effect size estimates were taken from recent meta-analyses examining the effects of suboptimal breastfeeding on intestinal infectious disease and acute respiratory infection incidence and mortality [1,23]. These values are summarized in Table 2. Where separate data were not available for mortality and incidence, we assumed the mortality effect size applied equally to incidence.

**Table 2:** Effect size estimates used in disease burden calculations

**Exposure**	**Age affected**	**Effect sizes (95% confidence interval)**			
		**Diarrhea incidence**	**Diarrhea mortality**	**Pneumonia incidence**	**Pneumonia mortality**
Predominant breastfeeding	0 to 5 months	1.26 (0.81 to 1.95)	2.28 (0.85 to 6.13)	1.79 (1.29 to 2.48)	1.75 (0.48 to 6.43)
Partial breastfeeding	0 to 5 months	1.68 (1.03 to 2.76)	4.62 (1.81 to 11.76)	2.48 (0.23 to 27.15)	2.49 (1.03 to 6.04)
No breastfeeding	0 to 5 months	2.65 (1.72 to 4.07)	10.52 (2.79 to 39.60)	2.07 (0.19 to 22.64)	15.13 (0.61 to 373.84)
Discontinued breastfeeding	6 to 23 months	2.18 (1.14 to 4.16)	2.18 (1.14 to 4.16)	-	-

Suboptimal breastfeeding has been associated with many short-term and long-term health outcomes, and the inclusion criteria defined for the GBD 2010 were used to determine which outcomes to include. These criteria state that a risk-outcome pair must: 1) be of likely importance to disease burden or policy based on previous work; 2) have sufficient data and methods to enable estimation of exposure distributions by country; 3) have sufficient evidence for causal effects based on high-quality epidemiological studies in which the findings were unlikely to be caused by bias or chance; and 4) have evidence to support generalizability of effect sizes [2]. Late initiation of breastfeeding was not included in these attributable burden estimates because of possible confounding in the three observational studies associating late initiation with an increased risk of neonatal mortality. Infants too weak or ill to breastfeed are more likely to die. Additionally, we were unable to determine if there are risks associated with late initiation that are independent of the risks associated with nonexclusive breastfeeding during the neonatal period. Other potential outcomes were not included in this study because of limitations of the existing evidence (diabetes [24], meningitis [25]) or little importance to global disease burden estimates or policy (eczema [11,26]). There is consistent evidence linking suboptimal breastfeeding with childhood and adult obesity [27]; however, this risk-outcome pairing was excluded from this analysis because obesity itself is considered a risk factor rather than a health outcome with associated disease burden. Only developing countries were included in this analysis because the effect size estimates used in this analysis were taken from studies done in developing countries, and they are not thought to be generalizable to developed countries where pathogen exposure levels are much lower.

We calculated population attributable fractions (PAFs) for death and disability due to non-exclusive and discontinued breastfeeding using the following formula:

$$\mathrm{PAF}=\frac{\sum_{i=1}^{n}P_{i}RR_{i}-\sum_{i=1}^{n}P{'}_{i}RR_{i}}{\sum_{i=1}^{n}P_{i}RR_{i}}$$

Where *Pi* is the prevalence of breastfeeding group *i*, *RRi* is the relative risk of death/disability for group *i*, and *P’i* is the prevalence of breastfeeding group *i* in a counterfactual population.

We computed the attributable burden by multiplying PAFs for death and disability by the underlying cause-specific mortality and morbidity from the GBD 2010 [20,28].

All analyses were conducted in Stata version 11 and Python version 2.4. Code is available by request.

## Results and discussion

### Trends in prevalence

**Figure 1:**
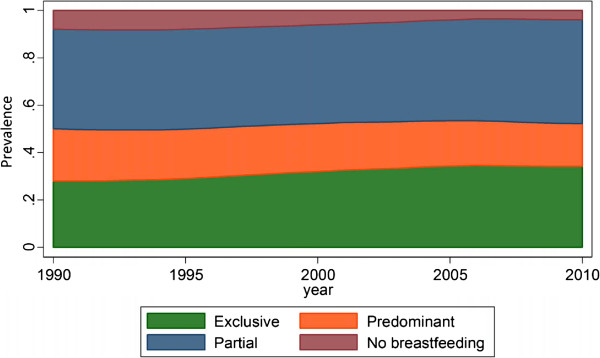
Global trend in breastfeeding behaviors in children under six months, 1990 to 2010.

Globally, progress towards the WHO recommendations on breastfeeding was very limited between 1990 and 2010. Figure 1 shows the small increase in exclusive breastfeeding prevalence among children less than six months old. In 1990, 27.9% of children less than six months old were exclusively breastfed compared to 34.2% in 2010. Between 1990 and 2010, a slight decrease was seen in the global continued breastfeeding prevalence between the ages of six and eleven months (75.6% in 1990 to 72.5% in 2010). A noticeable decrease in the prevalence of timely initiation of breastfeeding (41.5% in 1990 to 32.0% in 2010) was evident at the global level. Globally, the most progress was observed for continued breastfeeding between the ages of 12 and 23 months where prevalence increased from 31.9% in 1990 to 59.2% in 2010.

Behind these global trends, there was considerable variation in both levels and trends by country. Supplementary Table 1 [see Additional file 1] lists the country-specific prevalence estimates for each breastfeeding indicator in 1990 and 2010. In 2010, estimates of exclusive breastfeeding prevalence vary from 3.5% in Djibouti to 77.3% in Rwanda. Estimates of timely breastfeeding initiation (6.3% in Malawi to 58.0% in Chad) and continued breastfeeding (13.4% in Qatar to 95.5% in Gambia among children 6 to 11 months old; 17.1% in Qatar to 93.8% in Burundi among children 12 to 23 months old) also show considerable variation across countries.

**Figure 2:**
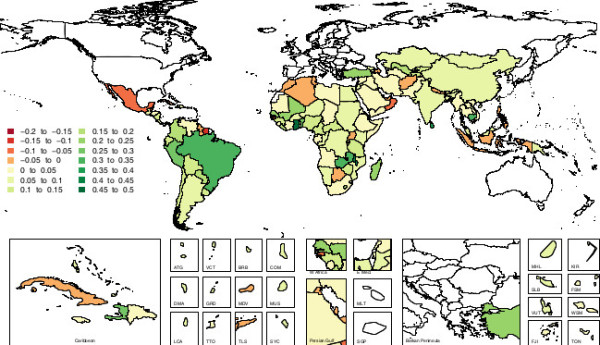
Map of absolute change in exclusive breastfeeding prevalence in developing countries among children younger than six months of age, 1990 to 2010.

**Figure 3:**
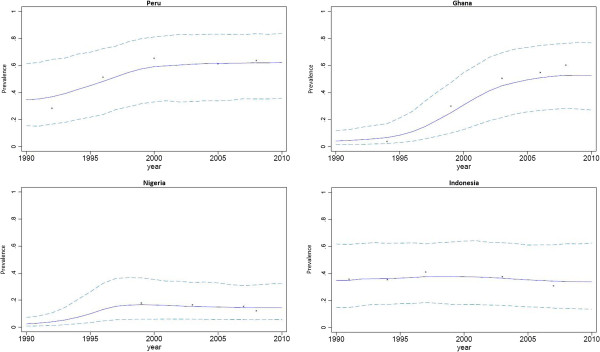
Exclusive breastfeeding prevalence trends in selected countries, 1990 to 2010.

Similarly, estimates of the change in breastfeeding prevalence between 1990 and 2010 showed high levels of variation at the country level. Figure 2 shows the absolute change in exclusive breastfeeding prevalence between 1990 and 2010 for each country in this analysis. Looking closely at these country level variations, several countries are notable and illustrative for their trends in breastfeeding prevalence. Figure 3 shows exclusive breastfeeding prevalence in four countries: Peru, Ghana, Nigeria and Indonesia. Ghana and Peru are notable for increases in exclusive breastfeeding prevalence observed over relatively short periods of time, and they are representative of a group of several countries that had successful and well-documented breastfeeding promotion efforts during the period of analysis. Nigeria and Indonesia are notable for their relatively constant prevalence of exclusive breastfeeding. Relatively flat or decreasing trends characterized nine of the ten most populous countries in the developing world: China, India, Indonesia, Pakistan, Nigeria, Bangladesh, Mexico, the Philippines, and Vietnam. Brazil was the only large nation where a marked increase in exclusive breastfeeding prevalence was observed between 1990 and 2010.

Some patterns observed in the prevalence rates indicate that there may be identifiable cultural factors influencing breastfeeding behaviors in certain regions, which may represent opportunities for successful interventions. For example, Cote d’Ivoire and Ghana, two West African neighbors, both had low prevalence of exclusive breastfeeding and very high prevalence of predominant breastfeeding in 1990. There appears to be a regional factor leading women to use breastfeeding as the primary source of nutrition for children, but also giving water or tea to infants at very high rates. By 2010, Ghana’s successful breastfeeding promotion efforts increased the country’s exclusive breastfeeding prevalence by 47% and decreased the predominant breastfeeding prevalence by 33%. Ghana now has one of the world’s highest prevalence rates of exclusive breastfeeding. Over the same time period, Cote d’Ivoire’s exclusive breastfeeding prevalence increased by only 2% and more than half of all infants younger than six months continue to be predominantly breastfed. Cote d’Ivoire continues to have one of the lowest prevalence rates of exclusive breastfeeding in the developing world. Looking at these data alone, it not possible to determine what caused these hugely divergent trends, but uncovering the drivers behind these trends could catalyze improvements in breastfeeding practices.

### Inequalities

**Figure 4:**
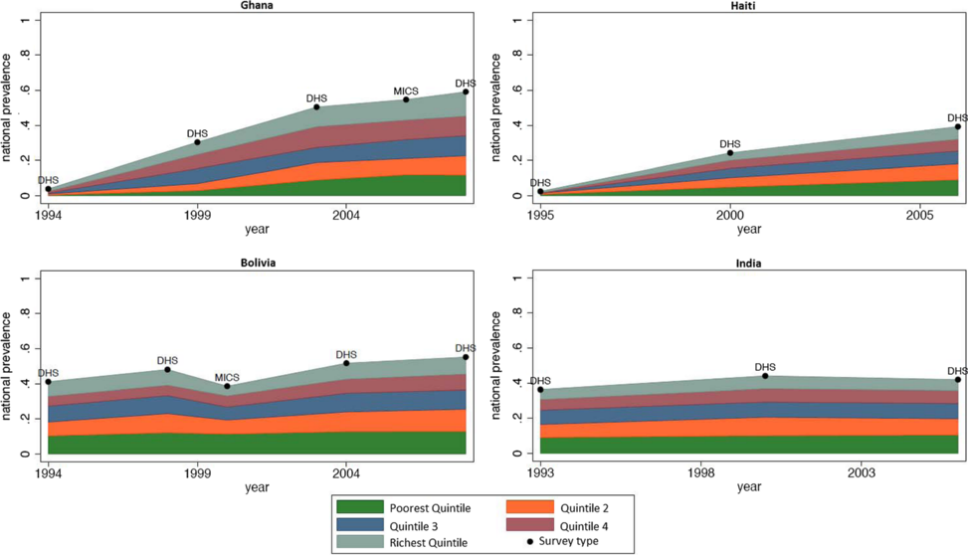
Breastfeeding prevalence by income quintile in selected countries.

The inequalities analysis was restricted to the 33 countries with three or more data points where breastfeeding information could be linked at the individual level with wealth data. Of these countries, thirteen had three years of data and twenty had four or more years of data. Figure 4 shows the exclusive breastfeeding prevalence by quintile in Ghana, Haiti, Bolivia and India, four countries that were representative in terms of geographic distribution and prevalence trends.

Three of these countries, Ghana, Haiti and Bolivia showed increases in exclusive breastfeeding prevalence over the period of analysis with a constant rate of increase observed across all wealth quintiles. The trends seen in Ghana were similar to patterns seen in Benin, Kenya, Mali, Malawi, Tanzania, the Central African Republic and Zambia, other African countries where increases in exclusive breastfeeding prevalence were measured. Haiti and Bolivia demonstrated this same pattern and were representative of the patterns seen in Colombia and Peru, the two other Latin American countries included in this portion of the analysis.

One of the dominant trends seen in the country-level analysis was constant prevalence over time in many of the most populous countries in the developing world. Of the 10 most populous developing countries, five—India, Indonesia, Vietnam, Nigeria and Bangladesh—had sufficient data to be included in this portion of the analysis. The data presented for India in Figure 4 are representative of the pattern seen in each of these countries - the constant prevalence over time seen at the country level was reflected in constant prevalence across all of the wealth quintiles.

There were select countries where varying rates of change were observed between wealth quintiles. Madagascar’s increases in breastfeeding prevalence appeared to be concentrated in the highest wealth quintiles, and Togo’s increases appeared to be concentrated in the upper half of the wealth distribution. However, the dominant trend observed across the countries was constant rates of change in prevalence across all wealth quintiles. This finding is consistent with the mechanisms of breastfeeding promotion efforts that are frequently implemented at the community level and are less dependent on health infrastructure than other child health interventions such as vaccination campaigns.

### Attributable Burden

Globally, suboptimal breastfeeding accounted for 544,817 (95% confidence interval 338,453 to 775,077) deaths and 47.5 million (29.9 million to 67.5 million) DALYs in 2010. Among children younger than five years old, this represents 8.0% of all deaths and 7.6% of all childhood DALYs. There has been significant progress in reducing this burden over the last two decades. From 1990 to 2010, the absolute DALYs attributable to suboptimal breastfeeding decreased by 56.9% globally. As a proportion of total child DALYs, suboptimal breastfeeding decreased from 10.8% to 7.6%. These global changes are due to underlying changes in child morbidity and mortality, as well as changes in breastfeeding prevalence.

**Figure 5:**
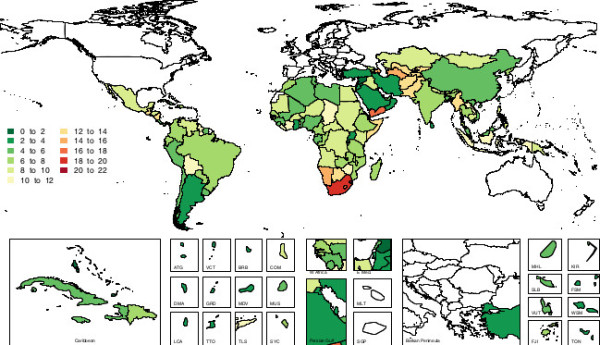
**Map of disease burden due to suboptimal breastfeeding in 2010, as measured by percent of a country's total child DALYs attributable to suboptimal breastfeeding.** DALYs, disability adjusted life years.

Beneath the global trends, there are large variations at the country level in estimates of disease burden attributable to suboptimal breastfeeding. Figure 5 shows a map of the percent of each developing country’s total child DALYs that are attributable to suboptimal breastfeeding. There are some obvious regional patterns, for example the Southern Sub-Saharan Africa region stands out as having the largest percent of attributable DALYs in 2010, specifically in Swaziland, South Africa and Lesotho. Other regions have disparate patterns by country, with large contrasts seen within North Africa / Middle East where Yemen has among the highest percent of DALYs attributable to suboptimal breastfeeding (16.9%) and Syria, Qatar, Palestine, Iran, Saudi Arabia and Bahrain have among the lowest (<3%).

Across countries, the disease burden attributable to suboptimal breastfeeding in 2010 ranged from a low of 65 DALYs in Antigua and Barbuda to a high of 10.6 million DALYs in India. The proportion of total DALYs attributable to breastfeeding was 7.6% at the global level and as high as 20.2% in Swaziland. Table 3 shows the top 10 countries with the highest absolute DALYs and the highest proportion of DALYs attributable to suboptimal breastfeeding in 1990 and 2010. Absolute DALYs are a function of population so the highest absolute DALYs attributable to suboptimal breastfeeding are seen in many of the most populous countries in the developing world: India, Pakistan, Nigeria, Indonesia and China. The DALYs attributable to breastfeeding as a percent of a country’s total DALYs shows the fraction of disease burden that is due to suboptimal breastfeeding. The only country that appears in the top 10 list for absolute and relative burden in 2010 is Pakistan.

**Table 3:** Top 10 countries with the highest absolute DALYs and the highest proportion of DALYs attributable to suboptimal breastfeeding in 1990 and 2010

**Absolute DALYs (thousands)**					**Percent of total country DALYs (%)**			
**Rank**	**1990**		**2010**		**1990**		**2010**	
1	25,730	India	10,581	India	26.6	Nicaragua	20.2	Swaziland
2	11,113	China	4,544	Pakistan	24.7	Turkmenistan	18.0	South Africa
3	6,551	Pakistan	3,105	Nigeria	23.2	Brazil	17.2	Lesotho
4	6,233	Nigeria	2,915	Democratic Republic of the Congo	22.4	Azerbaijan	16.9	Yemen
5	4,832	Indonesia	1,600	Indonesia	22.0	Mexico	15.7	Turkmenistan
6	4,052	Brazil	1,481	Ethiopia	20.3	Swaziland	14.9	Azerbaijan
7	4,010	Bangladesh	1,252	China	20.0	Tajikistan	14.3	Namibia
8	2,773	Ethiopia	1,218	Afghanistan	19.5	South Africa	13.6	Pakistan
9	2,612	Egypt	1,002	Burkina Faso	19.5	Yemen	13.5	Tajikistan
10	2,199	Democratic Republic of the Congo	935	Niger	19.4	Egypt	13.3	Myanmar

DALYs, disability adjusted life years.

**Figure 6:**
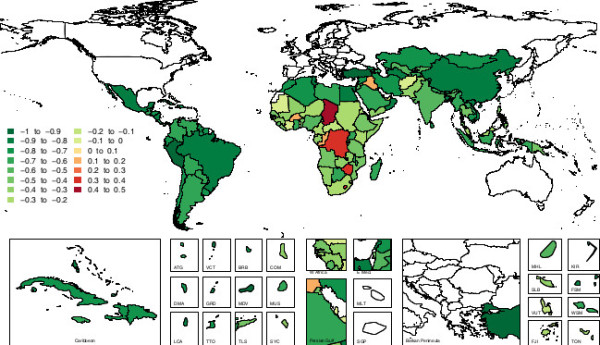
Percent change in DALYS attributable to suboptimal breastfeeding, 1990 to 2010.

At the global level there were large decreases in attributable deaths and DALYs. Figure 6 shows a map of the percent change in DALYs attributable to suboptimal breastfeeding by country from 1990 to 2010. Most countries appear to have a decrease in attributable DALYs, which may be due to improved adherence to breastfeeding standards, changes in population, or reductions in the underlying child mortality and morbidity. Despite widespread decreases, 5% of developing countries had an increase in absolute DALYs attributable to suboptimal breastfeeding between 1990 and 2010 (Chad, Lesotho, Zimbabwe, the Democratic Republic of the Congo, Burkina Faso, Congo, Iraq).

**Figure 7:**
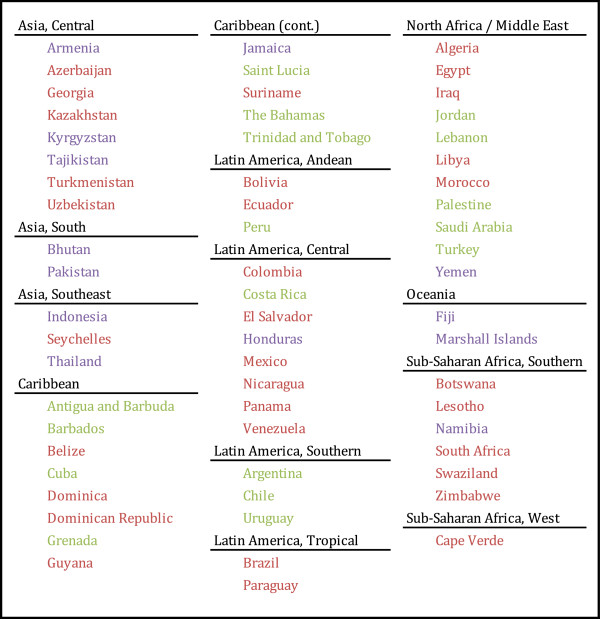
**Countries where suboptimal breastfeeding is the leading child risk factor in 1990 or 2010.** Countries in green are those where suboptimal breastfeeding was the top child risk factor in 1990 only; countries in purple are those where suboptimal breastfeeding was the top child risk factor in 2010 only; countries in red are those where suboptimal breastfeeding was the top child risk factor in 1990 and 2010.

Despite significant global progress, suboptimal breastfeeding was the second largest child risk factor in 1990 and remained the second largest child risk factor in 2010, ranking behind underweight (malnutrition). In 2010, suboptimal breastfeeding was the leading child risk factor in nearly one-third of all developing countries (42 of 137 countries). This is down from 49 countries in 1990. Figure 7 shows the countries where suboptimal breastfeeding was a leading child risk factor in 1990 or 2010. The majority of countries on the list in 1990 remain so in 2010; however, with changes in breastfeeding prevalence, exposure to other risk factors, and patterns of child death and disability, some countries have shifted on or off the list over the last two decades. Other notable child risk factors include underweight (malnutrition), iron deficiency, zinc deficiency and second-hand smoke exposure. Across all developing countries, suboptimal breastfeeding ranks higher than water and sanitation in terms of attributable DALYs.

*The Lancet’s* 2013 Maternal and Child Nutrition Series (MCNS) found 804,000 deaths attributable to suboptimal breastfeeding in 2011, representing 11.6% of total deaths of children younger than five years old [6]. This is a higher figure than the 544,817 attributable deaths we estimate in this analysis for 2010. This analysis estimated global breastfeeding prevalence rates similar to those in the MCNS analysis. In 2010, we estimated exclusive breastfeeding at 34.2%, predominant breastfeeding at 17.7% and partial breastfeeding at 36.1%. The MCNS analysis estimated 30.4%, 25.8% and 35.4%, respectively. However, this may mask significant differences in country-level breastfeeding prevalence estimates. The relative risks used for diarrhea incidence and mortality in children 6 to 23 months old were similar across the two analyses. The MCNS analysis also included pneumonia as an outcome associated with discontinued breastfeeding based on relative risks from a study in press. No published, statistically significant relative risks for discontinued breastfeeding and pneumonia were available during this analysis so pneumonia was not included as an outcome associated with discontinued breastfeeding. Finally, as stated in the MCNS publication, differences in attributable deaths may stem from differences in cause-specific deaths between the UN estimates used by MCNS and the GBD estimates used in this analysis.

### Limitations

Further research into breastfeeding patterns is needed to improve future estimates of breastfeeding exposure and associated risks. There are very few available data on breastfeeding behaviors in several regions, most notably the Middle East and Central Asia. Our spatial-temporal regressions made the best possible estimates from the existing data, but data limitations invariably result in limitations to the estimates. The only way to improve future prevalence estimates in these regions is to improve the quality and availability of the data. Additionally, the current body of breastfeeding literature provides little evidence on the effects of HIV prevalence and antiretroviral therapy scale-ups on breastfeeding behaviors. There are several theories and plausible mechanisms about possible interactions, but little data currently exist on what is happening in this area of critical public health importance. Data limitations prevented exploration of these interactions as part of this study.

Additionally, the lack of epidemiological evidence linking late initiation of breastfeeding with health outcomes inhibited our ability to interpret fully the estimates of associated disease burden. The exclusion of breastfeeding initiation from these estimates of attributable disease burden prevents an informed discussion about a significant component of current WHO breastfeeding recommendations. We recognize the ethical challenges of producing more accurate estimates of the effects of breastfeeding initiation; however, more needs to be done to utilize existing data and research opportunities to produce effect size estimates for timely initiation of breastfeeding that are independent of the effects of exclusive breastfeeding during the neonatal period and free from confounding.

The limited understanding of the ways breastfeeding’s biological pathways of effect interact with various other child health interventions also limits our ability to interpret these results. It is widely accepted that breastfeeding has strong immunopotentiation effects and may reduce exposure to pathogens during infancy [29,30]. However, the interactions between breastfeeding and other child health interventions, such as water and sanitation interventions and immunizations, are poorly understood. Developed countries were excluded from this analysis because we did not feel the existing effect size estimates could be generalized to settings with significantly lower rates of pathogen exposure. However, the developed/developing dichotomy masks considerable variation within each category, and these differences likely result in significant variation across space and time in the morbidity and mortality effects of breastfeeding. Resulting discrepancies can be seen in the variation between breastfeeding trends and attributable disease burden trends presented here, but these discrepancies cannot be analyzed and explained until improved evidence on the interactions of breastfeeding’s health promoting effects is made available.

Finally, in the GBD 2010, risk factor attributable burden is calculated for each risk factor independently, holding other factors unchanged [2]. For example, suboptimal breastfeeding and underweight both affect diarrhea and pneumonia and when we analyze each risk independently a single child death from diarrhea may be attributed to each risk factor. However, we can still compare across risks, acknowledging that each is independent and child deaths cannot be summed across risk factors.

## Conclusions

Breastfeeding promotion remains an intervention of enormous public health potential. It is widely regarded as one of the most cost-effective child health interventions currently available and does not require extensive health system infrastructure. These characteristics, along with the large disease burden associated with suboptimal breastfeeding, indicate that breastfeeding promotion has the potential to improve child health outcomes.

Another implication of these results is that breastfeeding promotion has the potential to simultaneously promote child health equity. Countries such as Ghana and Haiti demonstrate the trend seen in many other countries, where breastfeeding prevalence increased at a constant proportion across all wealth quintiles. When this finding is considered alongside data demonstrating that diarrhea and pneumonia-related disease burden is concentrated in lower wealth quintiles [22,31], the multiplicative relationship of these indicators suggests that a 10% increase in breastfeeding prevalence in all quintiles will result in a larger absolute reduction in disease burden in the lowest wealth quintiles. This, taken jointly with the evidence demonstrating that other critical maternal and child health interventions exhibit significant wealth-related inequities [32,33], suggests that breastfeeding promotion programs are better positioned to reduce wealth-related child health inequities compared to other maternal and child health intervention programs.

The overall disease burden from suboptimal breastfeeding has more than halved from 1990 to 2010, but there is still a considerable gap between current breastfeeding prevalences and WHO recommendations. It is important to examine what information can be learned by exploring the drivers behind observed trends. Studies that have taken a closer look at breastfeeding interventions in Ghana, Madagascar, Bolivia and Brazil provide illustrative case studies of how community-based breastfeeding promotion, legislative efforts and media campaigns can be implemented to rapidly improve breastfeeding behaviors [10,15,34]. Similarly, studies in countries such as Mexico have identified social and political factors that may be associated with decreasing breastfeeding prevalence [35].

Breastfeeding promotion is a unique type of child health intervention because it requires less health infrastructure to be implemented successfully. It requires more understanding of the complex cultural ideas surrounding breastfeeding behaviors. The diverse country and regional trends reported in this study call attention to the need for greater understanding of the drivers of changes in breastfeeding behavior so that future money spent on breastfeeding promotion can achieve greater results. Additionally, we hope that the data presented in this paper will enable policy makers to focus intensified efforts on breastfeeding promotion in countries such as Cote d’Ivoire that currently have a very low prevalence of optimal breastfeeding behaviors, but may have the potential to respond rapidly to well-targeted interventions.

## Abbreviations

CI: Confidence interval; DALY: Disability adjusted life year; DHS: Demographic and Health Survey; GBD: Global Burden of Disease; GDP: Gross domestic product; GPR: Gaussian process regression; HIV: Human immunodeficiency virus; MCNS: Maternal and Child Nutrition Series; MICS: Multiple Indicator Cluster Survey; OLS: Ordinary least squares; PAF: Population attributable fraction; PAPFAM: Pan-Arab project for family health; RHS: Reproductive health survey; RR: Relative risk; WHO: World Health Organization.

## Competing interests

The authors declare that they have no competing interests.

## Authors’ contributions

TJR identified the data sources, contributed to all parts of the analysis, and wrote the first draft of the manuscript. EC performed the estimation of attributable disease burden, produced several tables and figures, and assisted with writing and editing the manuscript. EG conceptualized the project and guided the data analysis and manuscript writing. All authors read and approved the final manuscript.

## Authors’ information

Thomas J Roberts is a medical student at Stanford University School of Medicine, Stanford, CA, USA. Emily Carnahan is a Monitoring and Evaluation Associate, PATH, Seattle, WA, USA. Emmanuela Gakidou is a Professor at the Institute for Health Metrics and Evaluation (IHME), University of Washington, Seattle, WA, USA.

## Pre-publication history

The pre-publication history for this paper can be accessed here:

http://www.biomedcentral.com/1741-7015/11/254/prepub

## Supplementary Material

Additional file 1: Table S1 Estimates and 95% uncertainty intervals for 187 countries for 1990 and 2010 of: early initiation of breastfeeding (<24 hours), exclusive breastfeeding (0 to 5 months), predominant breastfeeding (0 to 5 months), partial breastfeeding (0 to 5 months), continued breastfeeding (6 to 11 months), and continued breastfeeding (12 to 23 months). Click here for file
