# Supplementary material for: Can breastfeeding promote child health equity? A comprehensive analysis of breastfeeding patterns across the developing world and what we can learn from them
Source: BMC Med. 2013 Dec 4;11:254. doi: 10.1186/1741-7015-11-254 (PMC3896843; doi:10.1186/1741-7015-11-254)
Supplement: Additional file 1: Table S1 — Estimates and 95% uncertainty intervals for 187 countries for 1990 and 2010 of: early initiation of breastfeeding (<24 hours), exclusive breastfeeding (0 to 5 months), predominant breastfeeding (0 to 5 months), partial breastfeeding (0 to 5 months), continued breastfeeding (6 to 11 months), and continued breastfeeding (12 to 23 months). [file 1741-7015-11-254-S1.docx]

**Supplementary Table 1.** Estimates and 95% uncertainty intervals for 187 countries for 1990 and 2010 of: early initiation of breastfeeding (<24 hours), exclusive breastfeeding (0-5 months), predominant breastfeeding (0-5 months), partial breastfeeding (0-5 months), continued breastfeeding (6-11 months), and continued breastfeeding (12-23 months).

| **Country** | **Early initiation (<24 hours)** | | **Exclusive breastfeeding (0-5 months)** | | **Predominant breastfeeding (0-5 months)** | | **Partial breastfeeding (0-5 months)** | | **Continued breastfeeding  (6-11 months)** | | **Continued breastfeeding  (12-23 months)** | |
| --- | --- | --- | --- | --- | --- | --- | --- | --- | --- | --- | --- | --- |
|  | **1990** | **2010** | **1990** | **2010** | **1990** | **2010** | **1990** | **2010** | **1990** | **2010** | **1990** | **2010** |
| Afghanistan | 33.8 (24.0 - 44.1) | 31.1 (22.5 - 41.1) | 26.1 (9.1  - 53.4) | 25.0 (8.5  -  49.2) | 40.5 (26.7 - 54.2) | 40.6 (26.5 - 55.4) | 28.3 (17.7 - 40.3) | 26.0 (16.2 - 38.2) | 89.0 (75.9 - 96.2) | 82.5 (65.8 - 93.2) | 78.9 (61.8 - 90.7) | 76.2 (58.0 - 89.4) |
| Algeria | 23.1 (16.0 - 31.9) | 17.7 (12.2 - 25.0) | 16.8 (5.3  - 37.4) | 13.6 (4.3  - 30.0) | 30.1 (19.1 - 42.4) | 25.0 (14.2 - 38.5) | 40.1 (26.7 - 53.7) | 40.0 (25.8 - 55.9) | 76.2 (55.3 - 90.7) | 60.8 (37.2 - 81.6) | 62.0 (40.3 - 80.8) | 45.8 (24.6 - 68.5) |
| Angola | 39.5 (29.3 - 50.1) | 26.7 (18.2 - 36.6) | 14.3 (4.6  - 32.4) | 17.2 (5.4  - 39.4) | 34.9 (24.3 - 46.8) | 27.8 (18.2 - 38.3) | 48.4 (36.5 - 60.2) | 50.4 (37.7 - 61.9) | 93.6 (86.1 - 97.9) | 84.4 (66.9 - 94.5) | 91.4 (81.9 - 96.8) | 80.3 (62.3 - 91.5) |
| Antigua and Barbuda | 33.7 (25.0 - 44.7) | 27.1 (19.1 - 37.1) | 14.5 (4.1  - 34.3) | 21.3 (6.5  - 45.3) | 6.2 (3.0  - 10.8) | 3.6 (1.7  - 6.3) | 63.0 (44.1 - 77.8) | 60.3 (42.0 - 77.3) | 49.9 (27.4 - 72.6) | 51.9 (29.1 - 74.1) | 33.9 (15.4 - 55.2) | 36.0 (17.4 - 57.4) |
| Argentina | 27.4 (19.4 - 37.5) | 23.9 (16.9 - 32.5) | 45.6 (19.7 - 73.2) | 50.8 (23.2 - 77.6) | 8.8 (4.5  - 15.7) | 5.7 (2.9  - 10.2) | 43.1 (30.2 - 58.9) | 45.9 (33.6 - 62.3) | 69.7 (47.6 - 86.9) | 68.4 (45.7 - 85.1) | 55.2 (31.2 - 76.7) | 49.5 (28.6 - 71.9) |
| Armenia | 30.2 (21.6 - 40.5) | 27.1 (18.9 - 36.7) | 33.6 (13.2 - 60.0) | 41.0 (16.0 - 68.6) | 33.7 (21.6 - 46.3) | 24.8 (14.4 - 36.7) | 27.9 (17.1 - 39.5) | 28.2 (17.4 - 41.4) | 65.0 (42.1 - 83.5) | 58.7 (35.4 - 78.3) | 39.4 (18.8 - 61.2) | 35.8 (17.9 - 57.8) |
| Azerbaijan | 49.7 (38.8 - 60.8) | 42.3 (31.4 - 54.0) | 19.2 (5.9  - 40.6) | 16.0 (5.5  - 35.7) | 36.4 (24.7 - 49.3) | 32.0 (20.8 - 43.6) | 36.4 (25.0 - 49.1) | 40.8 (28.2 - 54.2) | 59.7 (34.7 - 80.1) | 53.2 (32.4 - 73.9) | 44.6 (23.9 - 66.8) | 37.6 (19.3 - 59.8) |
| Bahrain | 20.2 (13.8 - 28.1) | 19.4 (13.1 - 26.9) | 29.5 (10.0 - 55.9) | 28.2 (10.6 - 53.5) | 12.8 (6.5  - 21.3) | 6.3 (3.0  - 11.0) | 46.8 (29.4 - 61.9) | 50.1 (32.3 - 67.9) | 60.4 (34.5 - 80.8) | 54.7 (30.5 - 76.4) | 66.6 (44.9 - 83.8) | 52.1 (30.0 - 73.5) |
| Bangladesh | 47.0 (36.4 - 57.4) | 18.8 (12.8 - 26.6) | 40.8 (17.5 - 69.0) | 46.5 (21.9 - 71.5) | 18.2 (10.3 - 29.5) | 12.9 (7.0  - 20.3) | 36.1 (23.6 - 51.7) | 39.0 (27.2 - 52.8) | 94.0 (87.5 - 97.7) | 90.8 (81.2 - 96.5) | 90.2 (79.5 - 95.8) | 85.8 (72.6 - 93.8) |
| Barbados | 33.4 (24.3 - 44.1) | 25.7 (17.9 - 35.2) | 15.1 (4.6 - 35.6) | 21.1 (6.8 - 46.2) | 7.8 (3.7 - 13.7) | 5.3 (2.3 - 9.7) | 53.0 (33.8 - 69.4) | 54.2 (31.3 - 71.2) | 39.5 (19.7 - 62.7) | 45.9 (23.9 - 69.1) | 29.2 (13.0 - 49.5) | 33.0 (15.4 - 56.1) |
| Belize | 26.2 (17.9 - 35.7) | 23.8 (16.1 - 32.5) | 20.0 (6.5  - 43.3) | 13.0 (4.1  - 29.6) | 23.4 (13.3 - 35.9) | 12.0 (6.1  - 19.4) | 39.0 (24.6 - 54.3) | 56.8 (40.0 - 70.9) | 69.6 (46.6 - 86.3) | 55.6 (31.4 - 76.7) | 51.9 (30.2 - 73.8) | 40.0 (20.5 - 61.0) |
| Benin | 37.7 (28.1 - 48.1) | 21.4 (14.8 - 29.4) | 13.5 (3.9  - 30.8) | 38.8 (16.2 - 66.4) | 37.9 (25.8 - 50.7) | 23.8 (14.4 - 36.4) | 45.3 (32.0 - 57.2) | 35.8 (23.3 - 50.3) | 94.5 (88.7 - 98.0) | 93.0 (85.7 - 97.4) | 88.6 (77.4 - 95.6) | 84.6 (71.0 - 93.5) |
| Bhutan | 41.6 (30.9 - 52.7) | 26.5 (18.2 - 35.6) | 39.6 (14.5 - 66.6) | 45.3 (21.0 - 72.1) | 21.3 (11.2 - 33.9) | 20.0 (10.9 - 32.0) | 30.5 (18.5 - 46.8) | 27.0 (16.8 - 39.6) | 87.0 (73.1 - 95.1) | 74.5 (53.4 - 88.9) | 74.3 (53.8 - 88.9) | 61.2 (39.7 - 79.9) |
| Bolivia | 36.7 (27.1 - 46.8) | 17.4 (11.8 - 24.7) | 42.4 (20.3 - 67.8) | 54.0 (27.8 - 78.6) | 9.5 (5.1  - 16.3) | 6.5 (3.3  - 11.5) | 39.7 (26.8 - 54.4) | 36.2 (24.2 - 51.7) | 82.2 (69.8 - 91.1) | 84.4 (70.2 - 93.0) | 61.8 (43.5 - 78.9) | 66.7 (45.7 - 83.8) |
| Botswana | 26.8 (19.0 - 36.3) | 10.9 (7.3  - 15.4) | 27.5 (10.1 - 54.8) | 22.8 (7.9  - 46.2) | 25.7 (14.9 - 37.5) | 12.2 (6.2  - 20.9) | 41.0 (27.5 - 55.4) | 52.4 (36.6 - 67.3) | 85.5 (71.3 - 94.4) | 74.4 (52.9 - 89.4) | 68.7 (49.1 - 83.6) | 39.8 (20.8 - 61.5) |
| Brazil | 36.3 (26.4 - 47.0) | 30.3 (21.6 - 40.1) | 5.5 (1.7  - 13.0) | 35.8 (14.9 - 63.9) | 16.6 (9.0  - 25.9) | 8.5 (4.1  - 15.7) | 52.3 (36.2 - 66.1) | 34.4 (18.9 - 52.1) | 43.5 (25.2 - 62.4) | 39.2 (18.5 - 63.7) | 33.6 (18.5 - 51.1) | 29.6 (14.4 - 49.4) |
| Burkina Faso | 51.4 (40.0 - 61.8) | 41.7 (31.4 - 52.4) | 2.5 (0.7  -  6.4) | 11.0 (3.5  - 25.7) | 77.8 (70.0 - 84.8) | 62.8 (51.4 - 72.8) | 15.5 (9.5  - 22.7) | 22.4 (14.7 - 32.0) | 91.8 (84.2 - 96.6) | 94.0 (87.6 - 97.8) | 86.1 (74.6 - 93.9) | 89.0 (78.2 - 95.8) |
| Burundi | 24.3 (16.9 - 32.8) | 18.8 (12.9 - 26.3) | 67.3 (40.2 - 87.8) | 65.6 (38.5 - 86.5) | 13.8 (7.8  - 22.0) | 18.3 (10.6 - 28.7) | 14.3 (8.3  - 23.2) | 18.4 (10.8 - 28.5) | 92.3 (83.4 - 97.0) | 95.0 (88.2 - 98.3) | 89.9 (79.3 - 95.8) | 93.8 (86.8 - 97.8) |
| Cambodia | 71.3 (61.2 - 79.3) | 39.5 (29.9 - 50.2) | 13.3 (4.0  - 31.2) | 45.4 (20.7 - 72.4) | 62.8 (50.1 - 74.4) | 31.1 (19.6 - 45.2) | 20.6 (12.7 - 29.6) | 17.0 (9.3  - 26.6) | 94.3 (87.2 - 98.2) | 86.5 (73.1 - 94.8) | 85.7 (72.9 - 94.4) | 71.4 (52.0 - 86.9) |
| Cameroon | 45.1 (34.7 - 55.6) | 41.9 (30.9 - 53.3) | 6.5 (1.8  - 15.8) | 21.4 (7.9  - 45.2) | 42.3 (30.5 - 54.2) | 35.2 (22.9 - 48.1) | 47.6 (35.2 - 59.6) | 40.5 (28.4 - 53.1) | 90.6 (81.8 - 96.2) | 89.9 (79.8 - 96.1) | 76.9 (59.9 - 88.6) | 74.0 (55.9 - 87.4) |
| Cape Verde | 30.3 (21.4 - 40.9) | 28.3 (19.7 - 38.2) | 11.1 (3.4  - 25.1) | 40.5 (16.8 - 67.1) | 25.9 (15.2 - 37.6) | 12.0 (6.0  - 19.7) | 48.5 (33.7 - 61.9) | 35.1 (20.5 - 50.0) | 90.5 (79.2 - 96.7) | 79.8 (61.4 - 92.0) | 67.6 (46.2 - 85.3) | 66.8 (45.1 - 83.9) |
| Central African Republic | 39.5 (29.5 - 50.1) | 37.3 (27.5 - 47.9) | 5.3 (1.5  - 13.2) | 19.5 (6.8  - 41.0) | 42.0 (30.8 - 53.6) | 30.0 (19.1 - 42.4) | 48.5 (37.2 - 59.8) | 47.4 (35.1 - 59.6) | 92.5 (84.4 - 97.1) | 92.8 (85.2 - 97.4) | 85.8 (73.9 - 94.1) | 85.0 (70.0 - 94.1) |
| Chad | 63.5 (52.0 - 74.1) | 58.0 (47.3 - 68.8) | 3.7 (1.0 - 9.2) | 7.5 (2.1  - 18.6) | 60.5 (50.1 - 70.2) | 53.5 (41.2 - 64.1) | 32.4 (22.9 - 42.4) | 34.2 (24.2 - 45.2) | 91.4 (81.5 - 96.5) | 88.9 (76.8 - 96.0) | 84.7 (71.4 - 93.5) | 80.9 (62.6 - 91.9) |
| Chile | 28.3 (19.8 - 38.4) | 24.1 (16.7 - 33.1) | 48.3 (23.0 - 75.2) | 58.7 (29.9 - 82.1) | 8.3 (4.5  - 14.3) | 4.2 (2.1  -  7.6) | 41.2 (28.3 - 55.5) | 43.7 (31.6 - 57.7) | 66.3 (41.6 - 84.9) | 63.8 (38.0 - 83.4) | 50.6 (27.4 - 72.7) | 46.9 (24.7 - 68.4) |
| China | 42.1 (31.2 - 53.4) | 28.7 (19.8 - 38.1) | 28.7 (10.4 - 54.8) | 35.9 (14.3 - 63.4) | 17.7 (9.6  - 27.8) | 5.0 (2.2  -  9.3) | 39.5 (25.4 - 56.1) | 39.4 (22.2 - 57.2) | 74.9 (56.4 - 88.6) | 60.6 (35.8 - 80.2) | 55.0 (35.9 - 75.3) | 38.5 (19.6 - 60.5) |
| Colombia | 21.3 (14.7 - 29.2) | 19.7 (13.8 - 27.0) | 11.8 (3.9  - 26.6) | 31.6 (13.4 - 58.2) | 10.0 (5.6  - 16.2) | 10.0 (5.2  - 16.5) | 64.4 (50.0 - 76.1) | 51.8 (37.5 - 66.3) | 58.7 (40.2 - 73.9) | 71.7 (53.1 - 85.1) | 40.2 (24.3 - 57.6) | 52.7 (33.7 - 71.1) |
| Comoros | 31.0 (22.2 - 40.7) | 30.4 (21.4 - 40.7) | 6.6 (1.8  - 15.6) | 14.8 (4.3  - 32.4) | 23.0 (14.4 - 32.2) | 12.4 (6.8  - 20.2) | 64.4 (52.9 - 74.6) | 64.9 (50.7 - 77.4) | 83.9 (66.9 - 93.6) | 81.0 (64.5 - 92.3) | 68.3 (48.2 - 84.9) | 62.7 (39.6 - 81.0) |
| Congo | 30.9 (21.7 - 40.9) | 28.0 (19.9 - 37.5) | 11.9 (3.3  - 27.6) | 19.1 (5.8  - 40.4) | 30.1 (19.6 - 41.5) | 21.6 (12.5 - 33.0) | 55.0 (41.7 - 67.2) | 54.7 (40.1 - 68.0) | 90.1 (78.0 - 96.4) | 87.5 (74.6 - 95.2) | 72.5 (50.7 - 88.1) | 67.4 (47.6 - 82.8) |
| Costa Rica | 32.7 (23.5 - 43.5) | 24.5 (17.6 - 33.0) | 18.3 (5.3  - 40.4) | 26.6 (9.2  - 51.7) | 9.6 (5.0  - 16.2) | 6.6 (3.1  - 11.8) | 56.9 (39.5 - 72.5) | 54.1 (36.8 - 70.8) | 57.5 (32.1 - 79.6) | 59.1 (35.4 - 80.6) | 38.1 (19.1 - 60.6) | 43.4 (22.4 - 67.2) |
| Cuba | 34.5 (24.8 - 45.0) | 27.6 (19.2 - 36.9) | 33.9 (12.7 - 61.7) | 32.4 (12.4 - 57.0) | 10.2 (4.8  - 17.9) | 7.8 (3.9  - 13.8) | 41.3 (26.6 - 58.9) | 44.3 (29.5 - 59.9) | 49.7 (26.7 - 72.6) | 46.3 (23.5 - 70.0) | 31.6 (14.9 - 52.0) | 27.0 (12.1 - 46.3) |
| Côte d’Ivoire | 44.5 (33.6 - 55.3) | 46.5 (35.7 - 57.2) | 4.0 (1.1  - 10.3) | 5.9 (1.8  - 15.0) | 58.8 (48.3 - 68.9) | 55.2 (43.5 - 66.4) | 33.1 (23.6 - 43.7) | 34.5 (24.2 - 46.3) | 91.9 (83.4 - 96.9) | 90.8 (79.7 - 96.7) | 80.7 (66.6 - 91.3) | 81.1 (64.2 - 92.2) |
| Democratic Republic of the Congo | 24.1 (16.6 - 32.6) | 18.7 (12.7 - 26.0) | 26.1 (9.3  - 53.5) | 32.2 (12.6 - 58.5) | 30.4 (19.5 - 42.3) | 23.5 (13.7 - 34.4) | 42.6 (28.8 - 55.7) | 40.9 (28.3 - 54.5) | 94.3 (87.5 - 98.0) | 90.3 (80.4 - 96.1) | 86.8 (72.5 - 95.1) | 80.2 (64.6 - 91.1) |
| Djibouti | 10.8 (7.1  - 15.7) | 6.9 (4.4  - 10.3) | 5.0 (1.3  - 13.4) | 3.5 (1.0  -  9.0) | 31.0 (22.1 - 40.0) | 20.7 (12.6 - 29.5) | 57.9 (47.8 - 68.1) | 66.0 (53.4 - 76.3) | 78.1 (58.8 - 91.2) | 67.7 (44.7 - 85.8) | 48.3 (26.8 - 71.3) | 45.1 (24.8 - 67.8) |
| Dominica | 34.0 (24.8 - 43.9) | 26.5 (18.9 - 35.8) | 14.5 (4.0  - 33.1) | 21.4 (7.1  - 45.7) | 13.2 (6.8  - 22.3) | 8.2 (4.1  - 14.3) | 55.7 (38.5 - 70.0) | 56.0 (39.2 - 71.5) | 52.6 (27.7 - 75.5) | 55.3 (32.4 - 78.5) | 35.2 (17.1 - 57.6) | 38.4 (19.5 - 61.4) |
| Dominican Republic | 25.0 (17.3 - 33.9) | 24.7 (16.9 - 34.2) | 10.7 (3.4  - 23.6) | 13.4 (4.0  - 31.3) | 17.2 (9.8  - 26.9) | 8.2 (4.0  - 14.4) | 53.5 (38.9 - 66.6) | 58.8 (40.3 - 72.9) | 47.6 (29.9 - 66.4) | 45.5 (24.4 - 65.7) | 33.9 (19.0 - 50.4) | 32.6 (16.5 - 53.2) |
| Ecuador | 32.2 (22.8 - 41.5) | 23.5 (15.7 - 32.8) | 30.7 (12.1 - 55.4) | 45.5 (20.6 - 72.6) | 14.4 (7.3  - 23.0) | 13.6 (7.3  - 22.0) | 45.3 (32.0 - 59.5) | 39.3 (27.4 - 55.1) | 74.4 (55.8 - 88.3) | 82.5 (65.3 - 93.7) | 49.2 (29.5 - 69.0) | 60.4 (38.2 - 80.2) |
| Egypt | 27.2 (18.9 - 36.6) | 18.6 (12.6 - 25.4) | 40.0 (17.1 - 66.8) | 46.1 (22.1 - 72.5) | 20.2 (10.7 - 33.1) | 19.7 (11.3 - 31.0) | 31.2 (19.6 - 44.8) | 29.6 (19.2 - 41.7) | 87.2 (76.6 - 94.1) | 89.7 (79.0 - 95.9) | 76.2 (60.3 - 87.3) | 81.0 (65.4 - 91.3) |
| El Salvador | 56.7 (46.2 - 67.0) | 35.1 (25.7 - 45.4) | 15.8 (5.0  - 32.9) | 25.6 (9.5  - 48.2) | 27.7 (18.1 - 39.3) | 9.7 (5.4  - 16.7) | 45.7 (32.0 - 58.7) | 54.4 (38.8 - 69.6) | 76.6 (56.7 - 90.2) | 68.6 (45.2 - 85.6) | 68.3 (48.1 - 85.1) | 66.7 (45.6 - 83.3) |
| Equatorial Guinea | 35.6 (25.6 - 46.1) | 22.2 (15.7 - 30.7) | 25.2 (8.5  - 51.5) | 32.8 (12.3 - 59.6) | 33.5 (21.7 - 46.8) | 15.2 (8.7  - 23.6) | 36.6 (24.5 - 49.7) | 45.6 (31.2 - 60.0) | 93.0 (84.6 - 97.6) | 80.2 (61.2 - 92.5) | 77.9 (58.4 - 90.6) | 56.0 (32.9 - 77.5) |
| Eritrea | 30.7 (22.4 - 40.2) | 15.8 (10.6 - 22.5) | 51.6 (24.9 - 76.8) | 52.6 (26.6 - 78.1) | 32.0 (19.6 - 46.8) | 31.0 (19.0 - 46.0) | 13.1 (7.3  - 21.3) | 12.6 (6.9  - 21.0) | 93.7 (86.6 - 97.6) | 91.5 (81.9 - 97.0) | 87.6 (76.2 - 94.5) | 80.9 (63.2 - 92.5) |
| Ethiopia | 26.9 (18.6 - 36.6) | 20.2 (13.6 - 28.2) | 47.9 (22.3 - 74.7) | 51.9 (24.6 - 80.1) | 17.6 (9.4  - 28.7) | 13.7 (7.2  - 23.0) | 29.2 (17.3 - 43.8) | 28.3 (17.4 - 43.4) | 92.8 (84.8 - 97.3) | 85.8 (71.4 - 94.4) | 84.2 (70.3 - 93.4) | 73.4 (54.2 - 87.5) |
| Federated States of Micronesia | 42.6 (32.1 - 53.5) | 30.0 (20.6 - 40.8) | 41.6 (18.1 - 70.0) | 42.4 (17.9 - 72.3) | 4.8 (2.4  -  8.4) | 3.0 (1.4  -  5.4) | 49.8 (35.0 - 66.8) | 50.1 (35.7 - 67.3) | 88.8 (75.7 - 96.2) | 83.6 (67.3 - 93.7) | 77.5 (60.3 - 89.9) | 69.7 (48.1 - 86.5) |
| Fiji | 42.1 (32.2 - 53.5) | 28.9 (20.6 - 38.4) | 41.1 (15.5 - 69.4) | 42.8 (18.0 - 72.5) | 3.7 (1.8  -  6.7) | 2.3 (1.1  -  4.2) | 50.2 (34.7 - 68.1) | 50.5 (35.4 - 67.8) | 81.8 (63.8 - 93.4) | 78.1 (57.1 - 91.7) | 68.2 (46.7 - 84.9) | 63.1 (41.2 - 81.8) |
| Gabon | 33.5 (24.5 - 43.2) | 26.5 (18.8 - 36.2) | 6.3 (1.8  -  16.3) | 7.4 (2.0  - 18.5) | 28.9 (18.5 - 39.4) | 23.2 (14.4 - 34.0) | 58.8 (46.3 - 70.1) | 59.5 (46.2 - 71.7) | 84.9 (69.4 - 94.4) | 75.5 (54.5 - 90.7) | 50.9 (28.6 - 73.9) | 39.9 (19.1 - 62.4) |
| Georgia | 64.9 (54.4 - 74.7) | 52.3 (40.9 - 62.9) | 16.9 (5.3  - 35.7) | 23.0 (8.3  - 45.0) | 13.2 (6.3  - 23.0) | 10.0 (4.4  - 17.8) | 42.1 (24.8 - 59.1) | 39.4 (22.3 - 57.4) | 52.0 (28.8 - 75.7) | 47.8 (25.7 - 70.0) | 42.2 (24.7 - 63.3) | 36.8 (19.0 - 58.1) |
| Ghana | 44.0 (33.6 - 55.4) | 25.1 (17.4 - 34.1) | 4.7 (1.5  - 11.8) | 52.3 (27.1 - 76.7) | 53.7 (41.7 - 65.3) | 20.5 (11.9 - 31.8) | 38.3 (27.1 - 50.5) | 24.2 (15.7 - 35.8) | 93.4 (86.4 - 97.3) | 93.4 (86.6 - 97.5) | 82.7 (68.6 - 91.7) | 84.3 (69.8 - 93.6) |
| Grenada | 34.5  (24.7 - 45.3) | 26.6  (19.0 - 35.3) | 14.3  (4.1  -  32.5) | 21.1  (7.0  - 46.4) | 16.4  (8.9  - 25.8) | 10.3  (5.1  - 17.4) | 54.9  (39.2 - 70.0) | 53.9  (36.5 - 69.1) | 58.6  (35.2 - 78.8) | 56.1  (32.6 - 78.3) | 40.4  (20.4 - 64.2) | 39.1  (19.6 - 62.5) |
| Guatemala | 28.7 (20.6 - 38.2) | 19.2 (12.7 - 27.0) | 41.6 (16.7 - 69.4) | 51.3 (24.5 - 76.0) | 22.2 (12.9 - 35.3) | 15.5 (8.6  - 24.8) | 30.1 (19.3 - 44.1) | 28.8 (18.5 - 41.6) | 72.3 (53.9 - 86.7) | 78.5 (59.9 - 91.8) | 48.1 (29.6 - 67.3) | 64.1 (41.7 - 81.4) |
| Guinea | 47.1 (36.0 - 57.2) | 31.8 (22.9 - 42.7) | 11.5 (3.0  - 27.4) | 24.7 (9.1  - 48.8) | 60.3 (47.5 - 72.5) | 51.7 (37.4 - 64.9) | 25.2 (16.1 - 36.3) | 20.5 (12.6 - 30.5) | 89.5 (78.6 - 96.1) | 88.3 (75.7 - 95.8) | 91.0 (82.2 - 96.5) | 87.5 (75.9 - 94.9) |
| Guinea-Bissau | 42.1 (31.3 - 52.8) | 28.6 (20.3 - 38.8) | 30.8 (10.7 - 59.5) | 23.7 (9.4  - 46.5) | 19.2 (11.0 - 30.0) | 34.8 (22.8 - 47.2) | 43.6 (30.0 - 58.2) | 33.6 (22.1 - 45.7) | 91.6 (81.4 - 97.1) | 91.5 (81.7 - 97.0) | 92.3 (82.9 - 97.4) | 90.8 (81.3 - 96.3) |
| Guyana | 35.0 (25.6 - 45.0) | 29.0 (20.9 - 40.3) | 25.7 (8.8  - 50.9) | 25.1 (9.1  - 48.9) | 14.7 (7.8  - 23.4) | 7.6 (3.9  - 13.3) | 46.6 (31.2 - 61.9) | 52.7 (35.3 - 68.3) | 61.2 (38.4 - 80.9) | 64.4 (41.2 - 83.4) | 55.4 (32.8 - 75.9) | 58.4 (35.9 - 77.9) |
| Haiti | 55.7 (45.0 - 65.9) | 33.7 (24.4 - 44.0) | 4.9 (1.4  - 12.2) | 31.7 (12.7 - 58.6) | 18.7 (11.6 - 27.8) | 11.0 (6.1  - 18.4) | 71.9 (62.1 - 80.7) | 52.9 (38.0 - 67.2) | 85.8 (72.3 - 94.2) | 80.6 (62.0 - 91.9) | 70.0 (50.4 - 83.9) | 64.5 (43.1 - 83.3) |
| Honduras | 25.3 (17.7 - 34.6) | 16.3 (11.0 - 23.6) | 34.6 (14.2 - 59.7) | 34.8 (14.5 - 60.9) | 19.4 (10.9 - 29.8) | 9.0 (4.6  - 15.6) | 40.1 (26.6 - 54.9) | 48.4 (34.2 - 64.6) | 83.5 (68.0 - 93.7) | 75.5 (55.4 - 89.3) | 72.1 (51.7 - 86.8) | 62.4 (40.9 - 80.9) |
| India | 63.0 (52.8 - 73.2) | 51.0 (38.6 - 62.0) | 37.8 (15.9 - 65.0) | 43.2 (20.1 - 70.2) | 29.6 (18.1 - 44.1) | 21.9 (12.4 - 32.8) | 26.6 (15.8 - 39.6) | 29.0 (18.5 - 42.7) | 90.2 (79.9 - 96.0) | 85.6 (71.8 - 93.8) | 79.3 (63.2 - 91.0) | 70.2 (50.2 - 85.3) |
| Indonesia | 51.2 (40.4 - 62.5) | 43.7 (33.5 - 54.8) | 36.0 (15.0 - 61.8) | 35.0 (13.6 - 62.4) | 9.0 (4.7  - 15.5) | 6.8 (3.5  - 11.9) | 49.3 (35.2 - 64.8) | 51.9 (37.6 - 68.6) | 89.1 (79.6 - 94.9) | 84.0 (69.9 - 93.7) | 78.6 (64.0 - 88.7) | 73.5 (54.2 - 87.7) |
| Iran | 25.6 (18.1 - 34.6) | 20.4 (14.0 - 28.7) | 43.6 (19.0 - 71.8) | 44.2 (19.2 - 71.8) | 36.4 (23.9 - 52.2) | 27.4 (16.7 - 41.2) | 19.6 (11.7 - 30.0) | 22.5 (13.5 - 34.6) | 94.7 (88.7 - 98.1) | 85.8 (72.2 - 94.7) | 90.8 (81.4 - 96.6) | 79.4 (61.0 - 91.5) |
| Iraq | 24.6 (17.4 - 33.2) | 21.7 (14.5 - 30.4) | 24.4 (8.3  - 50.4) | 27.0 (9.6  - 53.2) | 31.2 (19.7 - 43.6) | 29.6 (18.7 - 42.3) | 35.9 (24.1 - 48.1) | 32.0 (19.7 - 44.9) | 76.6 (57.2 - 90.0) | 72.4 (50.8 - 88.4) | 62.4 (39.0 - 82.8) | 61.6 (39.4 - 81.0) |
| Jamaica | 31.0 (22.4 - 40.2) | 26.5 (18.5 - 35.9) | 15.1 (4.5  - 36.2) | 16.6 (5.4  - 35.1) | 17.7 (10.7 - 26.9) | 13.3 (7.2  - 20.8) | 59.2 (44.2 - 71.4) | 61.7 (47.7 - 74.3) | 62.9 (38.6 - 82.0) | 58.3 (32.9 - 80.0) | 52.5 (30.5 - 75.0) | 47.6 (24.6 - 69.3) |
| Jordan | 31.3 (22.2 - 40.8) | 26.7 (19.3 - 36.0) | 16.8 (5.7  - 36.0) | 23.2 (7.0  - 47.4) | 21.8 (13.3 - 32.0) | 18.2 (10.2 - 28.6) | 54.1 (41.2 - 66.4) | 48.6 (35.1 - 62.8) | 68.3 (48.6 - 84.3) | 66.3 (47.3 - 82.7) | 46.3 (28.3 - 65.5) | 44.4 (26.0 - 63.9) |
| Kazakhstan | 55.2 (44.1 - 65.9) | 43.9 (33.2 - 54.9) | 15.3 (4.6 - 35.7) | 21.2 (7.7 - 41.9) | 38.0 (25.5 - 50.7) | 33.1 (21.3 - 45.3) | 36.6 (24.4 - 49.4) | 37.3 (24.4 - 49.9) | 74.4 (54.9 - 88.6) | 74.3 (54.1 - 89.2) | 51.0 (30.3 - 71.9) | 52.9 (32.7 - 75.3) |
| Kenya | 21.3 (14.6 - 29.2) | 19.1 (13.1 - 26.3) | 15.7 (5.5  - 32.7) | 22.9 (8.0  - 45.5) | 21.8 (13.3 - 31.2) | 15.0 (8.5  - 23.8) | 58.8 (46.4 - 70.5) | 59.6 (46.0 - 72.3) | 90.4 (83.0 - 95.5) | 88.8 (77.8 - 95.4) | 76.7 (61.8 - 87.8) | 73.3 (54.4 - 87.3) |
| Kiribati | 44.5 (33.3 - 55.4) | 30.4 (21.7 - 39.7) | 40.2 (17.4 - 68.9) | 42.4 (17.1 - 70.7) | 5.0 (2.4  -  9.3) | 2.9 (1.4  -  5.4) | 49.7 (35.4 - 67.0) | 50.6 (36.2 - 67.7) | 88.2 (75.2 - 95.7) | 84.3 (68.6 - 94.0) | 76.9 (58.6 - 90.1) | 70.0 (47.9 - 86.5) |
| Kuwait | 21.1 (14.3 - 29.1) | 19.5 (13.3 - 27.0) | 11.6 (3.3  - 27.5) | 11.5 (3.3  - 27.3) | 11.5 (5.9  - 19.4) | 4.4 (1.8  -  8.4) | 53.6 (33.6 - 70.1) | 49.9 (27.3 - 70.1) | 50.0 (26.2 - 72.5) | 42.8 (20.3 - 65.8) | 32.2 (15.8 - 53.0) | 19.8 (8.1  - 36.9) |
| Kyrgyzstan | 35.7 (25.8 - 45.9) | 35.1 (25.7 - 46.0) | 25.1 (8.5  - 48.9) | 35.2 (12.7 - 62.7) | 26.9 (16.1 - 38.9) | 26.4 (15.9 - 38.4) | 44.7 (31.9 - 58.7) | 35.3 (23.4 - 48.7) | 83.4 (66.8 - 93.4) | 79.2 (60.8 - 91.5) | 67.6 (46.0 - 84.6) | 64.8 (45.5 - 82.5) |
| Laos | 50.3 (39.9 - 60.8) | 36.2 (26.2 - 46.6) | 18.3 (5.9  - 38.1) | 21.6 (7.3  - 43.4) | 23.3 (14.1 - 33.4) | 20.0 (11.5 - 30.3) | 54.3 (41.3 - 66.9) | 51.0 (38.4 - 64.6) | 95.6 (89.4 - 98.6) | 88.4 (75.7 - 95.8) | 87.3 (74.0 - 95.4) | 75.1 (55.6 - 88.8) |
| Lebanon | 29.8 (21.4 - 40.1) | 27.1 (18.7 - 36.2) | 16.7 (5.0  - 36.7) | 18.0 (5.3  - 40.4) | 28.1 (17.1 - 41.2) | 19.2 (9.2  - 31.4) | 35.6 (22.0 - 49.6) | 36.5 (20.7 - 51.6) | 57.6 (33.8 - 79.3) | 47.4 (24.1 - 70.1) | 42.8 (21.1 - 65.6) | 33.1 (15.9 - 53.9) |
| Lesotho | 33.7 (24.6 - 43.9) | 16.2 (11.3 - 22.5) | 17.1 (5.0  - 37.5) | 41.3 (18.6 - 65.3) | 25.2 (15.2 - 36.9) | 9.1 (4.8  - 15.1) | 53.9 (40.5 - 67.0) | 43.0 (30.8 - 57.8) | 92.2 (82.1 - 97.3) | 81.8 (66.5 - 91.0) | 85.6 (70.8 - 94.1) | 71.2 (55.0 - 85.4) |
| Liberia | 17.1 (11.5 - 24.0) | 16.0 (10.5 - 22.8) | 13.0 (4.3  - 29.8) | 29.8 (11.2 - 55.4) | 24.9 (15.5 - 35.3) | 15.0 (8.0  - 24.1) | 53.7 (40.0 - 66.4) | 52.2 (38.2 - 66.8) | 77.6 (58.4 - 91.2) | 90.8 (81.0 - 96.5) | 60.6 (38.2 - 79.3) | 71.2 (52.7 - 85.6) |
| Libya | 23.9 (16.4 - 32.8) | 20.0 (13.4 - 27.6) | 23.6 (7.9  - 47.1) | 25.3 (8.9  - 48.9) | 21.4 (12.3 - 32.8) | 16.5 (9.0  - 26.0) | 45.6 (31.8 - 60.2) | 46.0 (31.1 - 61.2) | 70.1 (48.6 - 86.3) | 68.0 (44.4 - 85.7) | 55.5 (32.8 - 76.8) | 53.5 (30.8 - 74.5) |
| Madagascar | 20.8 (14.3 - 28.7) | 11.6 (7.7  - 16.7) | 31.1 (12.6 - 57.1) | 54.0 (29.1 - 76.9) | 20.5 (11.5 - 32.8) | 13.9 (8.0  - 22.6) | 38.6 (25.0 - 53.6) | 29.0 (18.4 - 41.0) | 90.3 (81.9 - 95.7) | 92.6 (84.7 - 97.0) | 71.8 (53.5 - 85.9) | 81.0 (66.5 - 90.8) |
| Malawi | 9.9 (6.4  - 14.7) | 6.3 (4.1  -  9.6) | 5.0 (1.5  - 13.4) | 49.7 (24.1 - 76.0) | 26.0 (15.5 - 38.3) | 12.2 (6.6  - 20.5) | 60.8 (46.6 - 73.1) | 34.5 (23.3 - 49.5) | 89.0 (79.5 - 95.2) | 93.2 (84.6 - 97.3) | 76.3 (61.3 - 88.6) | 86.4 (73.1 - 94.4) |
| Malaysia | 41.1 (30.3 - 52.1) | 27.9 (19.2 - 37.7) | 25.6 (8.7  - 51.8) | 34.0 (12.6 - 63.9) | 13.9 (7.1  - 22.2) | 7.4 (3.5  - 13.5) | 49.7 (34.7 - 65.2) | 46.5 (30.6 - 64.2) | 81.1 (62.3 - 92.5) | 67.3 (43.7 - 85.5) | 63.4 (41.9 - 82.8) | 46.8 (25.8 - 68.9) |
| Maldives | 15.1 (10.0 - 21.5) | 10.7 (7.0  - 15.5) | 46.6 (19.9 - 74.1) | 45.4 (19.0 - 72.7) | 12.4 (6.2  - 20.8) | 8.2 (4.2  - 14.9) | 39.4 (26.0 - 55.4) | 42.5 (29.3 - 61.1) | 94.8 (87.6 - 98.2) | 82.5 (68.1 - 92.3) | 89.3 (78.3 - 95.9) | 70.9 (51.9 - 85.2) |
| Mali | 35.8 (26.7 - 46.6) | 20.5 (13.6 - 29.4) | 7.9 (2.3  - 18.7) | 30.8 (11.3 - 56.8) | 70.7 (59.5 - 79.2) | 54.5 (39.2 - 70.0) | 16.1 (10.0 - 23.5) | 10.3 (5.7  - 16.3) | 90.7 (82.2 - 96.0) | 90.7 (80.9 - 96.2) | 79.3 (63.8 - 90.2) | 80.1 (63.3 - 91.3) |
| Marshall Islands | 41.6 (31.4 - 52.6) | 29.5 (20.9 - 40.1) | 23.8 (7.9  - 50.1) | 31.2 (10.8 - 61.7) | 4.4 (2.2  -  7.9) | 3.2 (1.5  -  6.1) | 63.2 (45.8 - 78.8) | 57.5 (40.2 - 75.3) | 86.2 (70.8 - 95.0) | 83.0 (66.6 - 94.3) | 75.0 (56.8 - 89.6) | 70.1 (49.1 - 85.9) |
| Mauritania | 42.1 (32.0 - 53.1) | 29.7 (21.2 - 39.1) | 11.5 (3.2  - 26.7) | 13.9 (4.0  - 34.4) | 40.3 (29.0 - 52.3) | 34.9 (22.6 - 47.4) | 42.3 (30.9 - 53.6) | 41.9 (28.8 - 54.6) | 94.7 (88.0 - 98.3) | 88.5 (75.9 - 95.8) | 91.1 (81.4 - 96.9) | 82.2 (66.0 - 92.6) |
| Mauritius | 40.8 (29.7 - 51.8) | 27.5 (19.4 - 36.7) | 26.2 (9.1  - 53.7) | 34.0 (12.9 - 63.0) | 10.6 (5.4  - 18.2) | 6.0 (2.8  - 10.6) | 50.7 (34.0 - 66.8) | 46.7 (29.9 - 65.3) | 74.4 (52.3 - 89.4) | 62.7 (38.9 - 82.9) | 57.1 (34.9 - 78.5) | 44.8 (23.6 - 68.6) |
| Mexico | 42.1 (31.5 - 53.6) | 36.2 (26.0 - 47.0) | 21.7 (7.6  - 46.4) | 13.1 (4.3  - 28.4) | 5.0 (2.3  -  8.9) | 4.2 (1.9  -  7.6) | 48.5 (30.8 - 65.4) | 51.8 (30.6 - 69.3) | 54.4 (32.2 - 74.2) | 50.0 (27.6 - 71.6) | 37.2 (18.7 - 58.9) | 34.6 (17.4 - 55.5) |
| Mongolia | 44.1 (32.8 - 54.9) | 40.4 (30.3 - 52.2) | 59.5 (31.1 - 83.7) | 67.7 (41.5 - 88.2) | 8.5 (4.4  - 14.7) | 3.8 (1.9  -  6.7) | 32.1 (21.1 - 45.6) | 32.0 (21.5 - 43.7) | 89.5 (78.2 - 96.3) | 86.6 (72.0 - 94.9) | 80.4 (62.5 - 91.9) | 78.5 (61.1 - 90.5) |
| Morocco | 21.1 (14.5 - 28.9) | 14.2 (9.4  - 20.3) | 37.1 (15.5 - 63.9) | 32.5 (11.5 - 58.6) | 17.6 (9.6  - 27.6) | 12.6 (6.2  - 21.2) | 35.9 (23.0 - 50.2) | 42.8 (29.2 - 58.1) | 73.7 (56.3 - 86.7) | 67.7 (44.9 - 85.2) | 55.2 (35.3 - 74.0) | 51.7 (29.6 - 72.5) |
| Mozambique | 12.5 (8.4  - 18.1) | 6.7 (4.2  - 10.0) | 27.9 (9.8  - 54.8) | 34.5 (14.1 - 60.8) | 38.2 (24.4 - 52.2) | 33.0 (22.0 - 46.0) | 29.4 (19.0 - 41.8) | 27.8 (18.0 - 40.0) | 90.6 (79.0 - 96.5) | 85.1 (68.8 - 93.9) | 82.0 (67.2 - 92.4) | 71.3 (49.1 - 87.0) |
| Myanmar | 41.8 (31.2 - 53.8) | 30.0 (21.5 - 39.9) | 16.5 (4.9  - 38.6) | 19.4 (6.0  - 42.2) | 47.5 (35.3 - 58.6) | 36.0 (24.2 - 47.9) | 33.3 (23.0 - 44.7) | 38.9 (27.4 - 51.8) | 94.2 (87.4 - 98.0) | 88.0 (75.0 - 95.7) | 90.2 (78.9 - 96.3) | 82.2 (67.3 - 93.0) |
| Namibia | 20.6 (13.7 - 28.5) | 14.5 (9.5  - 21.5) | 13.0 (3.9  - 29.9) | 21.7 (7.2  - 45.3) | 25.3 (15.4 - 37.1) | 10.5 (5.6  - 17.0) | 55.4 (42.7 - 67.7) | 59.9 (44.2 - 73.8) | 80.4 (66.7 - 90.9) | 74.0 (53.7 - 88.3) | 64.2 (43.9 - 80.6) | 57.3 (36.7 - 76.1) |
| Nepal | 41.9 (31.4 - 53.1) | 22.5 (15.4 - 30.6) | 63.9 (35.3 - 84.7) | 59.3 (31.5 - 82.7) | 7.0 (3.5  - 12.5) | 11.9 (6.4  - 19.8) | 21.6 (13.0 - 33.8) | 24.9 (15.3 - 37.7) | 95.3 (90.1 - 98.3) | 92.1 (84.2 - 96.9) | 91.4 (83.5 - 96.7) | 86.8 (73.4 - 94.5) |
| Nicaragua | 35.8 (26.6 - 46.0) | 20.5 (14.3 - 28.3) | 18.6 (5.6  - 40.5) | 31.6 (12.1 - 59.3) | 14.7 (8.3  - 23.5) | 9.7 (5.0  - 16.4) | 53.2 (36.9 - 68.5) | 49.6 (34.6 - 65.7) | 64.6 (39.9 - 82.6) | 65.0 (43.2 - 84.3) | 45.4 (25.3 - 66.3) | 50.2 (27.8 - 72.1) |
| Niger | 59.7 (49.0 - 70.0) | 40.9 (30.8 - 52.0) | 0.8 (0.2  -  2.1) | 7.8 (2.3  - 18.6) | 60.4 (49.7 - 70.5) | 59.3 (49.4 - 69.5) | 35.1 (25.3 - 45.1) | 29.4 (20.4 - 39.0) | 91.3 (83.3 - 96.4) | 92.8 (85.0 - 97.4) | 82.3 (68.7 - 91.7) | 86.2 (72.8 - 94.1) |
| Nigeria | 39.8 (30.1 - 50.3) | 34.9 (25.6 - 45.3) | 2.9 (0.8  -  7.2) | 15.7 (5.7  - 32.4) | 23.6 (15.5 - 32.7) | 36.7 (25.2 - 48.3) | 69.8 (60.0 - 78.5) | 43.2 (31.3 - 55.9) | 89.5 (80.3 - 95.6) | 88.9 (78.7 - 95.6) | 72.6 (54.1 - 86.1) | 76.9 (60.0 - 88.9) |
| North Korea | 38.7 (29.1 - 49.6) | 28.3 (20.0 - 38.2) | 31.3 (11.0 - 60.2) | 37.3 (15.0 - 64.5) | 15.2 (8.3  - 24.6) | 9.4 (4.6  - 16.0) | 43.4 (29.4 - 59.5) | 40.0 (24.9 - 56.7) | 76.4 (55.1 - 90.5) | 70.8 (50.0 - 86.7) | 85.9 (71.7 - 94.3) | 82.5 (66.1 - 92.9) |
| Oman | 20.1 (13.5 - 28.3) | 19.6 (13.2 - 27.9) | 58.6 (30.8 - 82.9) | 49.3 (22.3 - 77.1) | 14.4 (8.5  - 22.5) | 8.4 (4.3  - 14.4) | 38.1 (27.9 - 49.7) | 40.5 (27.1 - 55.0) | 84.8 (69.1 - 94.0) | 64.8 (39.9 - 83.9) | 92.3 (84.0 - 97.0) | 82.8 (68.0 - 93.3) |
| Pakistan | 43.4 (33.0 - 54.8) | 35.7 (25.8 - 45.5) | 18.7 (6.0  - 39.1) | 21.2 (7.0  - 43.8) | 30.3 (16.9 - 45.4) | 31.2 (18.4 - 46.4) | 36.3 (22.2 - 51.4) | 33.7 (20.5 - 49.0) | 83.9 (69.7 - 93.0) | 78.1 (58.7 - 90.4) | 69.3 (50.6 - 84.0) | 65.7 (45.7 - 82.3) |
| Palestine | 20.7 (14.0 - 28.9) | 19.7 (13.2 - 27.7) | 25.2 (8.6  - 51.8) | 26.1 (8.8  - 51.1) | 26.1 (15.0 - 39.0) | 21.5 (12.7 - 33.4) | 42.4 (29.5 - 57.0) | 44.1 (28.9 - 59.8) | 81.4 (63.6 - 93.2) | 81.8 (61.9 - 92.9) | 65.2 (43.3 - 83.4) | 63.9 (42.9 - 82.7) |
| Panama | 32.4 (22.6 - 42.1) | 24.4 (17.3 - 33.4) | 18.0 (5.2  - 41.1) | 27.4 (9.7  - 56.2) | 11.6 (5.8  - 20.1) | 7.3 (3.5  - 12.6) | 55.0 (37.0 - 69.9) | 54.3 (38.9 - 71.1) | 57.6 (34.6 - 79.1) | 62.5 (38.2 - 83.3) | 38.6 (18.3 - 61.1) | 45.0 (24.1 - 69.6) |
| Papua New Guinea | 42.8 (32.1 - 54.0) | 28.9 (19.9 - 38.3) | 57.0 (30.0 - 82.8) | 63.8 (36.1 - 86.7) | 6.5 (3.2  - 11.4) | 4.5 (2.2  -  7.8) | 37.9 (26.9 - 52.7) | 36.0 (25.5 - 49.5) | 92.2 (81.9 - 97.3) | 87.5 (74.1 - 95.6) | 85.4 (70.5 - 94.4) | 77.9 (58.4 - 90.1) |
| Paraguay | 29.0  (20.9 - 38.2) | 20.5  (14.3 - 28.3) | 5.0  (1.5  -  12.6) | 24.6  (9.2  - 46.2) | 49.3  (37.4 - 60.9) | 18.8  (10.9 - 28.6) | 34.5  (24.6 - 44.9) | 43.6  (29.7 - 58.0) | 64.2  (43.4 - 81.1) | 64.4  (41.8 - 82.7) | 37.9  (20.5 - 56.8) | 38.5  (19.9 - 58.7) |
| Peru | 26.8 (18.8 - 36.3) | 13.7 (9.1  - 19.8) | 35.6 (15.5 - 61.4) | 61.4 (35.7 - 83.7) | 7.7 (3.9  - 13.4) | 4.2 (2.1  -  7.3) | 45.4 (30.3 - 62.2) | 32.2 (21.6 - 46.0) | 80.5 (66.6 - 90.2) | 87.0 (74.1 - 94.5) | 62.7 (44.1 - 78.3) | 67.0 (48.0 - 83.1) |
| Philippines | 32.8 (23.3 - 42.8) | 28.4 (20.5 - 38.5) | 35.7 (13.7 - 66.2) | 34.6 (13.8 - 60.3) | 16.6 (8.4  - 27.5) | 17.4 (9.6  - 28.8) | 30.8 (17.8 - 46.8) | 32.0 (20.4 - 47.4) | 72.8 (52.0 - 88.4) | 64.7 (45.0 - 82.3) | 54.5 (32.2 - 74.4) | 49.3 (29.7 - 68.7) |
| Qatar | 21.4 (14.6 - 30.2) | 19.9 (14.0 - 27.3) | 11.7 (3.0  - 27.8) | 11.1 (3.1  - 27.2) | 4.8 (1.9  -  9.1) | 0.3 (0.1  -  0.9) | 50.4 (26.6 - 72.0) | 14.5 (4.6  - 32.4) | 40.3 (18.5 - 63.6) | 13.4 (4.6  - 27.3) | 47.6 (26.1 - 69.5) | 17.1 (7.0  - 32.7) |
| Rwanda | 25.9 (17.9 - 36.2) | 15.3 (10.2 - 21.5) | 67.0 (41.2 - 86.2) | 77.3 (53.2 - 92.9) | 3.3 (1.6  -  6.2) | 2.6 (1.3  -  4.8) | 23.6 (14.8 - 35.2) | 17.0 (10.7 - 26.1) | 92.4 (84.8 - 96.7) | 93.5 (86.5 - 97.4) | 86.4 (74.2 - 93.9) | 84.7 (71.5 - 92.8) |
| Saint Lucia | 34.1 (24.9 - 44.8) | 26.4 (18.6 - 36.1) | 14.8 (3.9  - 32.4) | 21.0 (7.1  - 44.3) | 11.5 (6.2  - 18.7) | 7.3 (3.6  - 13.0) | 58.7 (41.8 - 74.4) | 57.3 (39.9 - 74.4) | 56.5 (33.2 - 79.1) | 55.8 (32.0 - 77.6) | 39.9 (21.1 - 60.7) | 39.3 (19.4 - 62.2) |
| Saint Vincent and the Grenadines | 34.3 (25.0 - 44.2) | 26.6 (18.6 - 36.2) | 14.5 (4.3  - 32.1) | 21.5 (6.3  - 49.9) | 14.7 (7.9  - 23.0) | 8.3 (4.0  - 14.4) | 55.2 (39.4 - 69.5) | 56.7 (38.9 - 72.8) | 56.7 (32.0 - 79.3) | 56.9 (32.7 - 79.5) | 40.3 (20.3 - 61.4) | 40.0 (20.5 - 62.5) |
| Samoa | 42.9 (31.8 - 53.5) | 29.2 (21.0 - 38.4) | 38.8 (15.3 - 65.5) | 40.7 (16.7 - 68.5) | 4.4 (2.1  -  7.9) | 2.9 (1.4  -  5.7) | 51.4 (36.4 - 69.1) | 51.3 (36.9 - 67.7) | 84.2 (67.5 - 94.4) | 80.0 (59.0 - 91.7) | 68.2 (46.3 - 85.2) | 61.9 (38.0 - 81.4) |
| Saudi Arabia | 22.4 (15.0 - 30.4) | 19.9 (13.5 - 27.6) | 27.1 (9.4  - 54.8) | 27.2 (8.4  - 54.8) | 16.3 (8.8  - 25.6) | 11.4 (6.0  -  18.8) | 49.9 (35.4 - 63.4) | 49.4 (32.6 - 64.7) | 75.0 (55.8 - 89.6) | 65.1 (39.7 - 83.8) | 65.4 (42.7 - 83.0) | 44.6 (23.1 - 66.6) |
| Senegal | 25.8 (18.1 - 34.3) | 22.0 (15.2 - 30.6) | 5.2 (1.4  - 12.8) | 25.6 (9.4  - 50.8) | 57.3 (46.7 - 67.5) | 44.3 (30.0 - 58.4) | 33.6 (23.7 - 44.6) | 25.7 (16.3 - 36.8) | 93.0 (86.3 - 96.9) | 92.5 (83.3 - 97.1) | 83.7 (70.7 - 92.3) | 82.4 (67.9 - 92.2) |
| Seychelles | 40.4 (30.4 - 51.1) | 28.2 (19.8 - 38.3) | 24.2 (8.1  - 49.6) | 33.7 (12.2 - 61.1) | 5.4 (2.6  - 10.0) | 2.5 (1.1  -  4.6) | 55.1 (36.6 - 71.0) | 51.0 (31.7 - 69.7) | 63.2 (39.3 - 83.7) | 58.3 (33.4 - 79.7) | 44.0 (22.3 - 65.6) | 42.4 (22.0 - 64.5) |
| Sierra Leone | 23.7 (16.2 - 32.8) | 17.8 (12.1 - 24.7) | 9.4 (2.5  - 22.4) | 13.5 (4.4  - 30.6) | 46.4 (34.8 - 58.2) | 29.8 (19.7 - 40.5) | 39.8 (29.2 - 51.2) | 52.2 (40.0 - 63.9) | 89.6 (78.1 - 96.2) | 89.5 (78.5 - 95.9) | 82.5 (66.0 - 92.6) | 76.9 (59.2 - 89.4) |
| Solomon Islands | 44.1 (33.2 - 55.0) | 30.8 (21.3 - 40.8) | 65.3 (37.0 - 86.1) | 70.9 (42.9 - 89.8) | 5.2 (2.6  -  9.2) | 3.6 (1.8  -  6.2) | 38.0 (27.4 - 51.9) | 35.6 (25.5 - 47.8) | 91.9 (82.8 - 97.1) | 86.5 (70.7 - 94.9) | 83.8 (69.1 - 93.4) | 75.8 (54.2 - 89.3) |
| Somalia | 35.0 (25.8 - 45.4) | 29.4 (20.2 - 39.4) | 9.4 (2.7  - 23.0) | 11.6 (3.3  - 27.2) | 16.4 (9.1  - 24.7) | 15.8 (9.2  - 24.0) | 63.9 (49.7 - 75.5) | 63.4 (50.1 - 75.7) | 75.8 (55.4 - 90.1) | 78.4 (58.9 - 91.1) | 46.2 (24.7 - 69.6) | 49.4 (27.0 - 71.1) |
| South Africa | 39.7 (29.9 - 51.2) | 29.1 (20.8 - 39.2) | 6.7 (1.8  - 17.4) | 9.5 (2.9  - 22.6) | 11.1 (5.7  - 18.1) | 6.9 (3.5  - 11.8) | 72.9 (59.9 - 83.2) | 70.4 (53.5 - 82.7) | 77.5 (57.7 - 91.1) | 67.1 (43.0 - 86.1) | 67.6 (43.3 - 84.8) | 57.6 (34.9 - 77.0) |
| Sri Lanka | 41.8 (31.1 - 53.3) | 28.0 (19.6 - 37.5) | 21.2 (7.2  - 43.6) | 61.1 (33.3 - 84.0) | 33.6 (22.6 - 46.3) | 18.8 (12.2 - 27.0) | 41.3 (29.6 - 53.4) | 34.2 (25.2 - 46.1) | 76.7 (59.4 - 89.7) | 71.5 (49.7 - 88.2) | 67.7 (46.5 - 83.7) | 89.3 (78.9 - 95.8) |
| Sudan | 28.2 (20.0 - 37.4) | 23.6 (16.4 - 33.6) | 14.8 (5.2  - 32.0) | 22.4 (6.6  - 46.3) | 34.9 (24.3 - 46.3) | 34.1 (21.5 - 48.4) | 46.1 (34.0 - 58.4) | 32.8 (21.6 - 45.0) | 89.3 (79.0 - 95.4) | 77.1 (57.8 - 90.7) | 73.3 (56.0 - 86.8) | 64.0 (41.8 - 80.8) |
| Suriname | 40.5 (30.4 - 51.5) | 27.2 (19.5 - 36.3) | 19.6 (6.7  - 42.5) | 5.4 (1.4  - 13.2) | 14.0 (6.6  - 22.0) | 12.9 (6.6  - 21.5) | 45.7 (27.5 - 62.6) | 52.8 (33.8 - 68.8) | 61.4 (38.2 - 81.8) | 54.6 (31.4 - 76.2) | 46.0 (22.9 - 68.6) | 38.2 (18.4 - 60.0) |
| Swaziland | 40.3 (30.2 - 51.3) | 21.9 (14.8 - 30.3) | 22.2 (7.3  - 46.3) | 31.2 (12.2 - 56.9) | 9.7 (5.0  - 17.0) | 5.1 (2.7  -  9.0) | 62.6 (47.0 - 76.8) | 56.9 (41.3 - 72.8) | 89.4 (77.8 - 96.3) | 81.6 (65.2 - 92.8) | 79.6 (61.1 - 91.3) | 68.6 (47.8 - 84.5) |
| Syria | 20.8 (14.2 - 29.1) | 14.3 (9.5  - 20.9) | 30.9 (11.3 - 57.6) | 31.1 (12.3 - 57.2) | 26.8 (16.2 - 38.8) | 21.5 (12.3 - 32.6) | 37.9 (25.7 - 51.6) | 37.2 (23.1 - 51.3) | 86.2 (71.2 - 94.8) | 76.2 (55.9 - 90.0) | 72.7 (53.9 - 87.2) | 59.1 (35.4 - 78.4) |
| São Tomé and Príncipe | 17.7 (12.2 - 25.1) | 16.8 (11.6 - 23.7) | 59.3 (30.9 - 82.9) | 49.5 (23.8 - 76.0) | 27.1 (16.7 - 41.1) | 13.4 (7.2  - 22.6) | 13.4 (7.6  - 21.1) | 31.4 (20.6 - 46.6) | 93.8 (86.5 - 97.9) | 93.2 (85.8 - 97.3) | 85.3 (72.0 - 94.5) | 79.4 (63.9 - 90.0) |
| Taiwan | 38.7 (28.4 - 49.4) | 27.6 (19.2 - 37.4) | 27.5 (9.3  - 52.7) | 34.4 (13.3 - 62.7) | 7.1 (3.4  - 12.6) | 1.7 (0.7  -  3.3) | 47.7 (28.9 - 65.4) | 40.5 (20.6 - 60.7) | 60.4 (37.1 - 79.6) | 47.1 (23.1 - 71.8) | 43.2 (21.2 - 65.7) | 34.8 (16.9 - 55.2) |
| Tajikistan | 49.0 (38.0 - 60.0) | 43.0 (32.0 - 53.9) | 18.7 (5.7  - 41.4) | 31.1 (11.4 - 57.5) | 44.1 (31.5 - 56.0) | 36.7 (24.9 - 50.7) | 35.0 (24.2 - 47.1) | 28.9 (18.8 - 41.8) | 89.8 (78.2 - 96.4) | 84.0 (68.9 - 93.5) | 76.5 (57.2 - 89.9) | 70.8 (51.2 - 86.0) |
| Tanzania | 16.7 (10.9 - 23.7) | 12.9 (8.4  - 18.7) | 21.5 (7.5  - 44.8) | 34.6 (14.0 - 61.0) | 25.6 (15.4 - 37.6) | 17.9 (10.3 - 29.6) | 47.5 (33.7 - 62.7) | 43.2 (29.8 - 59.1) | 92.1 (84.1 - 96.6) | 91.1 (80.7 - 96.7) | 84.8 (72.0 - 93.2) | 80.3 (64.0 - 91.7) |
| Thailand | 38.5 (28.7 - 49.3) | 27.1 (18.8 - 35.9) | 6.3 (1.8  - 15.2) | 7.0 (2.1  - 18.1) | 20.3 (12.3 - 30.7) | 19.5 (9.8  - 30.6) | 61.3 (48.5 - 73.1) | 46.1 (28.5 - 61.8) | 72.0 (50.4 - 87.2) | 49.3 (28.0 - 71.1) | 55.4 (33.7 - 74.1) | 32.3 (15.2 - 54.9) |
| The Bahamas | 30.8 (21.9 - 40.4) | 24.9 (17.0 - 33.6) | 15.2 (4.7  - 34.5) | 21.3 (6.6  - 44.4) | 3.4 (1.5  -  6.3) | 2.6 (1.2  -  4.6) | 54.3 (31.5 - 74.3) | 54.6 (33.0 - 73.9) | 37.3 (17.9 - 60.7) | 42.4 (19.5 - 65.6) | 27.0 (12.4 - 46.4) | 31.0 (14.1 - 53.0) |
| The Gambia | 35.6 (25.8 - 47.0) | 25.6 (17.8 - 35.1) | 26.2 (9.0  - 50.6) | 38.1 (15.9 - 67.0) | 42.8 (29.8 - 55.2) | 33.5 (20.7 - 47.4) | 29.6 (18.9 - 41.9) | 27.6 (17.0 - 41.5) | 96.7 (91.5 - 98.9) | 95.5 (89.6 - 98.5) | 96.6 (92.5 - 98.8) | 92.6 (84.4 - 97.2) |
| Timor-Leste | 7.0 (4.5  - 10.2) | 6.7 (4.4  - 10.1) | 53.3 (24.8 - 80.3) | 49.5 (23.2 - 76.4) | 7.7 (3.9  - 13.5) | 7.4 (3.7  - 13.7) | 36.7 (25.1 - 52.8) | 40.1 (27.5 - 55.6) | 87.5 (74.7 - 95.5) | 86.2 (73.7 - 94.2) | 54.8 (32.6 - 76.1) | 52.2 (30.2 - 73.4) |
| Togo | 50.7 (39.5 - 61.3) | 46.3 (35.8 - 57.3) | 13.0 (4.2  - 28.7) | 28.9 (10.7 - 54.6) | 62.9 (50.5 - 73.2) | 31.0 (19.3 - 43.9) | 21.1 (13.1 - 29.7) | 36.2 (24.3 - 49.6) | 93.3 (85.9 - 97.3) | 92.9 (84.1 - 97.7) | 84.9 (71.3 - 93.0) | 90.5 (81.4 - 96.5) |
| Tonga | 42.6 (32.2 - 53.8) | 29.4 (20.7 - 39.1) | 40.0 (17.6 - 68.7) | 41.4 (16.7 - 68.5) | 3.6 (1.8  -  6.6) | 2.2 (1.0  -  3.9) | 52.5 (37.1 - 69.0) | 53.6 (39.1 - 71.5) | 86.2 (72.1 - 94.9) | 83.5 (66.3 - 94.0) | 71.7 (51.6 - 87.7) | 68.0 (47.0 - 85.1) |
| Trinidad and Tobago | 35.1 (25.3 - 46.4) | 26.5 (18.4 - 36.0) | 12.8 (3.9  - 29.8) | 16.4 (5.0  - 35.7) | 11.5 (5.9  - 18.9) | 8.3 (4.1  - 14.1) | 56.5 (40.3 - 71.1) | 54.0 (35.5 - 70.9) | 43.2 (21.4 - 65.9) | 45.9 (24.9 - 68.2) | 33.7 (17.6 - 53.1) | 29.4 (13.1 - 49.4) |
| Tunisia | 24.7 (16.9 - 33.4) | 20.0 (13.3 - 28.0) | 22.8 (7.6  - 46.0) | 18.2 (5.9  - 36.8) | 29.5 (18.9 - 41.6) | 22.7 (13.1 - 34.8) | 38.3 (25.9 - 51.9) | 43.2 (28.9 - 58.2) | 64.2 (42.2 - 82.1) | 51.2 (28.6 - 75.0) | 49.7 (30.6 - 68.6) | 50.2 (28.9 - 71.0) |
| Turkey | 22.3 (15.4 - 30.6) | 23.3 (16.4 - 31.7) | 8.7 (2.5  - 21.5) | 31.0 (12.8 - 58.6) | 39.6 (27.8 - 51.7) | 24.6 (15.0 - 36.2) | 42.3 (30.4 - 54.6) | 40.7 (29.4 - 53.0) | 65.1 (44.1 - 82.0) | 65.9 (45.1 - 83.0) | 47.3 (29.4 - 66.3) | 47.8 (27.8 - 67.8) |
| Turkmenistan | 42.7 (31.3 - 54.2) | 40.0 (30.1 - 50.7) | 13.5 (3.9  - 31.8) | 14.0 (4.1  - 33.1) | 36.8 (24.6 - 50.1) | 27.3 (16.6 - 40.4) | 39.4 (27.3 - 52.8) | 41.9 (27.6 - 56.3) | 74.3 (53.9 - 89.6) | 61.8 (37.7 - 81.1) | 57.1 (35.9 - 76.8) | 45.3 (23.5 - 68.1) |
| Uganda | 15.1 (10.0 - 21.4) | 14.7 (9.8  - 20.6) | 57.1 (29.6 - 79.4) | 56.3 (30.3 - 80.1) | 8.9 (4.7  - 15.1) | 6.2 (3.2  - 10.8) | 27.3 (17.2 - 40.1) | 32.9 (20.8 - 46.6) | 88.0 (78.2 - 94.3) | 87.8 (75.0 - 95.2) | 71.3 (53.0 - 85.4) | 72.2 (52.1 - 86.7) |
| United Arab Emirates | 20.2 (13.6 - 27.7) | 19.5 (13.4 - 27.0) | 29.3 (10.4 - 55.3) | 28.9 (10.5 - 56.4) | 4.2 (1.9  -  7.7) | 2.6 (1.0  -  5.1) | 44.7 (24.6 - 63.4) | 40.2 (19.3 - 61.5) | 45.2 (22.2 - 68.9) | 38.2 (18.2 - 61.5) | 51.4 (28.1 - 73.9) | 35.4 (18.0 - 59.1) |
| Uruguay | 28.4 (19.8 - 38.0) | 24.2 (16.9 - 33.1) | 39.0 (15.3 - 66.2) | 53.6 (26.8 - 77.7) | 9.5 (4.8  - 16.5) | 5.6 (2.9  -  9.4) | 44.6 (30.6 - 60.6) | 44.4 (32.0 - 58.3) | 67.7 (43.9 - 86.2) | 67.6 (45.0 - 85.5) | 52.6 (29.8 - 73.4) | 49.4 (27.0 - 72.2) |
| Uzbekistan | 59.0 (47.2 - 69.8) | 57.7 (47.4 - 68.5) | 5.8 (1.5  - 14.2) | 21.4 (7.1  - 42.4) | 44.9 (33.9 - 55.7) | 38.0 (24.7 - 51.8) | 45.1 (33.8 - 56.4) | 35.7 (24.3 - 48.6) | 85.2 (70.6 - 94.1) | 84.9 (69.4 - 93.9) | 71.6 (51.4 - 86.7) | 71.5 (51.1 - 87.0) |
| Vanuatu | 42.9 (32.3 - 53.8) | 29.6 (20.7 - 39.4) | 34.4 (13.1 - 61.2) | 42.1 (16.1 - 70.6) | 3.0 (1.4  -  5.7) | 1.7 (0.8  -  3.3) | 54.4 (37.8 - 72.6) | 52.5 (38.1 - 71.6) | 90.7 (80.6 - 96.7) | 87.7 (74.4 - 95.3) | 82.6 (65.9 - 93.5) | 78.4 (60.3 - 91.0) |
| Venezuela | 33.0 (23.6 - 43.5) | 23.8 (16.5 - 32.7) | 5.1 (1.4  - 13.1) | 7.0 (2.0  - 17.5) | 13.1 (6.8  - 21.9) | 9.9 (5.2  -  17.4) | 65.0 (47.7 - 78.3) | 68.4 (50.9 - 80.7) | 57.1 (33.4 - 80.4) | 61.3 (36.1 - 81.5) | 38.8 (19.1 - 61.7) | 44.5 (22.7 - 65.1) |
| Vietnam | 21.8 (15.1 - 30.4) | 14.5 (9.7  - 20.7) | 20.1 (6.6  - 42.5) | 21.4 (7.7  - 44.9) | 37.7 (26.6 - 49.2) | 29.2 (18.9 - 40.0) | 41.5 (30.5 - 53.3) | 47.0 (33.5 - 59.7) | 96.7 (92.8 - 98.8) | 91.3 (81.9 - 97.0) | 84.0 (69.1 - 93.8) | 75.3 (56.8 - 88.5) |
| Yemen | 36.3 (27.1 - 48.0) | 31.9 (23.1 - 42.3) | 10.9 (3.7  - 25.1) | 13.8 (4.2  - 29.7) | 37.5 (25.0 - 50.8) | 29.0 (17.6 - 42.9) | 42.0 (28.6 - 55.4) | 39.2 (25.3 - 54.7) | 78.5 (63.6 - 89.8) | 74.7 (53.6 - 89.4) | 62.2 (43.3 - 79.3) | 59.4 (38.2 - 79.7) |
| Zambia | 11.9 (7.9  - 17.2) | 10.5 (7.0  - 15.3) | 14.0 (4.5  - 30.5) | 45.0 (20.2 - 72.6) | 38.7 (26.3 - 52.6) | 16.0 (8.9  - 26.1) | 41.5 (29.2 - 53.9) | 35.2 (23.2 - 50.9) | 90.7 (81.9 - 96.1) | 91.5 (82.9 - 96.5) | 82.0 (69.0 - 91.1) | 84.7 (71.3 - 93.2) |
| Zimbabwe | 18.6 (12.8 - 25.5) | 13.0 (8.5  - 18.6) | 11.6 (3.9  - 25.9) | 22.6 (7.0  - 46.4) | 28.3 (18.2 - 39.3) | 17.4 (9.9  - 26.7) | 57.6 (46.2 - 68.7) | 56.8 (43.1 - 70.6) | 92.0 (84.7 - 96.4) | 90.3 (80.8 - 96.4) | 82.5 (70.5 - 91.9) | 82.7 (68.7 - 92.3) |
